# A discourse and content analysis of representation in the mainstream media of the South African National Health Insurance policy from 2011 to 2019

**DOI:** 10.1186/s12889-023-15144-6

**Published:** 2023-02-07

**Authors:** Lynn Bust, Eleanor Whyle, Jill Olivier

**Affiliations:** Health Policy and Systems Division, School of Public Health, Faculty of Health Sciences, University of Cape Town, Cape Town, South Africa

**Keywords:** Discourse, Media, National health insurance, Low- and middle-income countries, Public health policy, Universal health coverage

## Abstract

**Background:**

Media is a crucial factor in shaping public opinion and setting policy agendas. There is limited research on the role of media in health policy processes in low- and middle-income countries. This study profiles South Africa as a case example, currently in the process of implementing a major health policy reform, National Health Insurance (NHI).

**Methods:**

A descriptive, mixed methods study was conducted in five sequential phases. Evidence was gathered through a scoping review of secondary literature; discourse analysis of global policy documents on universal health coverage and South African NHI policy documents; and a content and discourse analysis of South African print and online media texts focused on NHI. Representations within media were analysed and dominant discourses that might influence the policy process were identified.

**Results:**

Discourses of ‘health as a global public good’ and ‘neoliberalism’ were identified in global and national policy documents. Similar neoliberal discourse was identified within SA media. Unique discourses were identified within SA media relating to biopolitics and corruption. Media representations revealed political and ideological contestation which was not as present in the global and national policy documents. Media representations did not mirror the lived reality of most of the South African population. The discourses identified influence the policy process and hinder public participation in these processes. They reinforce social hierarchy and power structures in South Africa, and might reinforce current inequalities in the health system, with negative repercussions for access to health care.

**Conclusions:**

There is a need to understand mainstream media as part of a people-centred health system, particularly in the context of universal health coverage reforms such as NHI. Harmful media representations should be counter-acted. This requires the formation of collaborative and sustainable networks of policy actors to develop strategies on how to leverage media within health policy to support policy processes, build public trust and social cohesion, and ultimately decrease inequalities and increase access to health care. Research should be undertaken to explore media in other diverse formats and languages, and in other contexts, particularly low- and middle-income countries, to further understand media’s role in health policy processes.

**Supplementary Information:**

The online version contains supplementary material available at 10.1186/s12889-023-15144-6.

## Background

“*As individuals we are all influenced, our opinions shaped, reinforced and altered by our exposure to the media.*”

-- Macarro [1]

Media influences how people make meaning of the world around them and how they behave [2, 3].^1^ Media is a ‘social institution’ which supports the establishment of dominant discourses [4]. Discourses are diverse representations of social life which tend to simplify complex realities, and are inherently positioned by different social actors who see and represent social life in different ways [5, 6].^2^ This is based on the constructivist paradigm that meaning is socially constructed [7]. Media discourse has been described as a “public, manufactured, on-record, form of interaction” [8]. Discourses within media do not only reflect public perception, but also influence it [9].

Within public health, media plays a role in guiding public opinion, and influencing health behaviours, health service utilisation, and the formation of public policy [10]. The influence of media on health policymaking processes has been illustrated [9, 11, 12]. Decision-making by policy actors in the policy process is influenced by public perception, which in turn is influenced by media [13, 14]. Policy actors (in national settings) are defined as individuals and organisations who: i) develop formal policies (policymakers); ii) influence how policies are translated into practice (middle managers, health workers, patients, citizens); and iii) seek to influence the formal policy process (civil society and interest groups including private sector actors, actors within media, and advocacy organisations) [15]. Decisions made by these policy actors can influence what action is taken in the policy process, or whether any action is taken at all – influencing how the policy process unfolds [13].

While some media studies have been conducted in public health and in Health Policy and Systems Research (HPSR) [10, 16, 17], few studies have analysed the influence of media representations on health policy processes [10]. The limited available literature on the topic is largely restricted to high-income countries (HICs) and quantitative content analyses, which often lack explanatory capacity [10]. Studies which have analysed the role of media in health policy processes have highlighted the importance of context-specificity, understanding the influence of media within the social, economic and political context within which it has emerged [18, 19]. This study focuses on the influence of media representations on health policy processes in a low- and middle-income country (LMIC) setting. Specifically, the study explores the introduction of National Health Insurance (NHI) in South Africa (SA), answering the question: “*How is the South African NHI policy represented in mainstream print and online media, and how might this influence the policy process?”*.

The introduction of NHI in SA should be understood against a backdrop of a health system with massive inequalities between the public and private sector; rural and urban areas; high- and low-income groups; primary and tertiary levels of care; and geographic regions or provinces [20, 21]. These divides stem from the apartheid history of SA which enforced a system based on racial discrimination and repression [20]. The private health sector’s expansion was supported during apartheid [22], resulting in a historic skewing of resources towards the private sector which continues today (Table 1). The private sector in SA is substantial and is composed of a variety of actors.^3^ The most prominent actors in the private sector are private hospitals and ‘medical schemes’ (voluntary private health insurance) [23]. Three quarters of private hospital beds are owned by three large hospital groups [24]. These private facilities primarily serve the 16% of the population covered by medical schemes [25]. The other 84% of the population who are uninsured rely on the public sector which has approximately 50% of total expenditure on healthcare and 70% of the country’s usable hospital beds [25, 26].

**Table 1:** A brief overview of the SA private health sector compared to the public health sector [source: [20, 27–30]

	**Private Sector**	**Public Sector**
Components of the sector	Private health care providers Institutions that represent health providers Private health facilities (hospitals, laboratories) Funding mechanisms of private health services (medical schemes, life and short-term insurance) Traditional health practitioners	National Department of Health Nine provincial departments of health Three tiers of hospitals: tertiary, regional, district Primary health care system Local government responsible for preventive and promotive services
Funding and coverage	16% of population reliant on medical schemes which are voluntary private financing mechanisms Medical scheme coverage concentrated in top two income quintiles	84% of population reliant on public sector which is funded through taxes and out-of-pocket payments
Expenditure	Private sector expenditure in 2019 was 4.6% of GDP	Public sector expenditure in 2019 was 4.4% of GDP
National health facilities	225 acute hospitals More than 3/4 of private hospital beds owned by three large for-profit hospital groups (Mediclinic, Life Healthcare, and Netcare) Mainly located in major metropolitan areas	257 district hospitals, 49 regional hospitals, 21 tertiary hospitals
Doctor-to-patient ratio	1 doctor for 429 to 571 patients in private healthcare	1 doctor for 2 457 patients in public sector

One of the proposed mechanisms to address health system inequalities in SA is to implement NHI. The concept of NHI was first proposed in SA over 30 years ago in activist circles during apartheid [31]. It was more recently reintroduced by the post-apartheid ruling party, the African National Congress (ANC), and has since been kept on the political agenda [31, 32]. In 2009, the then Minister of Health (MoH), Aaron Motsoaledi, gave significant momentum to the process and NHI became more apparent in national discourse [33, 34]. SA is currently in the final stages of formulating the formal NHI policy [13]. New iterations of NHI policy documents have been released since 2011 (see appe file 1), culminating in the release of an NHI Bill in August 2019 (Table 2). The COVID-19 pandemic has been a reminder in many ways of the inequities in the current health system in SA [35]. It has somewhat delayed implementation of NHI, although work has continued in the background and is gaining momentum once again [36].

**Table 2:** Overview of the SA NHI Bill released in August 2019 [source: [37]

Purpose of the Bill	Establish an NHI fund that through mandatory prepayment aims to achieve sustainable and affordable universal access to quality health services by: ○ Serving as a single purchaser and payer to ensure equitable and fair distribution and use of health services ○ Ensuring the sustainability of funding for health services ○ Providing for equity and efficiency by pooling of funds and strategic purchasing from accredited and contracted health care service providers
Access to the fund	South Africans, permanent residents, refugees and inmates must register with the fund in order to be entitled to a package of benefits
Health facilities	The fund will purchase services on behalf of users at both private and public facilities that have been approved through an accreditation pro°°°cess
Governance of the fund	A Board that is accountable to the MoH will be established to govern the Fund
Financing	Money to pay for the fund will be provided from four named sources: ○ General tax revenue ○ Reallocation of medical scheme tax credit funds ○ Payroll tax (employer and employee) ○ Surcharge on personal income tax

The SA NHI Bill states that the purpose of NHI is to achieve Universal Health Coverage (UHC) [37]. UHC is defined by the World Health Organisation (WHO) as “ensuring that all people can use the promotive, preventive, curative, rehabilitative and palliative health services they need, of sufficient quality to be effective, while also ensuring that the use of these services does not expose the user to financial hardship” [38]. There is worldwide recognition of the need to progress towards UHC as a means to reduce financial impoverishment caused by health spending and increase access to key health services [39]. Many countries have implemented UHC policy reforms with different approaches and outcomes [40–42].

In SA, the NHI policy is profoundly political and contested at many levels [33, 43]. Research has shown that although there appeared to be public awareness of NHI in SA, there was limited public understanding of the knowledge and concepts surrounding it [44–46]. There was lack of public understanding and support for the core principles of universal pre-payment mechanisms such as cross-subsidisation [45]. Public perceptions of affordability, financial benefit, and quality of healthcare were found to be important drivers of support for NHI [44]. Private sector users were generally less supportive of NHI [44]. An online COVID-19 survey in 2020 revealed most urban respondents were in favour of NHI, but found that socio-economic status and education level influenced perceptions of NHI, with people with higher levels of education less likely to support it [47].

Media both reflects and shapes public perception [9], therefore it is crucial to investigate media (and the dominant discourses prevalent within media) as part of health policy processes [13]. In SA, media outlets are nominally independent from the state [48]. SA is characterized by a persistent class divide with an income inequality rate amongst the highest in the world [49]. SA media has been criticised for being targeted towards the elite, and is “still highly concentrated and not very diverse in terms of race and class” [48]. This study aims to describe the dominant discourses relating to the SA NHI within SA media to understand how this might influence the policy process.

## Method

This study is a descriptive mixed method media analysis. It was conducted in five sequential phases in an iterative manner during 2020 and 2021. Phase one was a scoping review of: a) media and health systems/HPSR; and b) UHC (focus on UHC reforms and SA NHI studies) from which key materials and themes were identified. The PRISMA extension for scoping reviews was utilised, and in total 401 articles were included (Fig. 3) [50]. Phase two applied Discourse Analysis (DA) on global policy documents (*n* = 15, Additional file 2) relating to UHC, tracing the themes from the scoping review, until saturation was reached. Phase three applied themes from both previous phases to conduct a DA of five iterations of the SA NHI policy documents released from 2011 to 2019.^4^ Phase four included the collection of media texts (*n* = 710) on SA NHI published during this time, and content analysis of these texts which provided an overview and description of them. Phase five combined the outcomes from the previous phases to guide a DA of the collected media texts which were then integrated into the results below. This process enabled an understanding of how the SA NHI is represented in media within the context of the previous phases, highlighting power and reproduction of social dominance. The analysis was primarily qualitatively driven, with the only phase that included quantitative methods being the content analysis in phase four which was incorporated into the analysis in the final phase.

Media texts containing the words ‘national health insurance’ or ‘NHI’ were collected over a selected period of two weeks before and after the release of each policy document (see Table 4).^5^ Texts were sourced through the SA Media database, Google, and PressReader. A systematic content analysis of these texts was conducted in phase four to provide a basic description of the data, including frequency of publication of texts and change over time, and identification of common topics and featured actors in the text [51].

DA was conducted in Phases 2, 3 and 5. DA consists of a broad category of methods and theories “investigating language in use and language in social contexts” [5]. Power and ideologies underly discourses represented within media, and analysing these discourses allows for the exploration of the connection between specific discourses and the broader social context and processes, including an analysis of power [5]. This study utilised ‘critical discourse analysis’, which is not a defined methodology, but rather a critical perspective taken during analysis [52]. Critical DA involves analysis of the way social-power abuse and inequality are enacted, reproduced, legitimated, and resisted by text and talk in the social and political context [52]. This is based on an analysis of power and its underpinning ideologies – examining the role that discourse plays in the production and reproduction of dominance by some social groups over others [53]. The critical perspective framework utilised in this study was guided by the work of Fairclough [5] and Van Dijk [52]. Fairclough’s framework for critical DA consists of three processes of analysis namely the object of analysis, the processes by which the object is produced and received, and the socio-historical conditions that govern this process [5]. This critical perspective was combined with the use of frame analysis as a particular DA methodology. Frames are a way of organising or understanding the overall discourse of a text [51]. This study utilised a qualitative frame analysis approach developed by Linström and Marais [54]. Qualitative frame analysis emphasises the cultural and political content of news frames, and how they “draw upon a shared store of social meanings” [55]. The findings from different components of the DA were integrated and compared in the final phases of the study.

A framework developed by Nixon and Power for increasing rigor in DA was followed which highlights that the goal of DA is to generate interpretive claims without the promise of objectivity [56]. This study combines both DA and content analysis which have epistemological differences. DA stems from interpretivism, whilst content analysis focuses more on causal explanation. However, if conducted in a rigorous manner, the two can complement each other. Methods to enhance trustworthiness include the application of theory, the use of quotations to substantiate argument, and triangulation of both data sources and methodological approaches. The researcher recognises that their own biases and perspectives (as a health policy and systems specialist who supports the principles of UHC and health equity) may have influenced the discourses identified and discussed.

## Results

This section highlights the discourses that emerged in global UHC and SA NHI policy documents, before discussing the content of the collected media texts. These results are then integrated into the description of the dominant discourses identified in media representations of the SA NHI below.

### Discourses in global UHC and SA NHI policy documentation

A DA was conducted on fifteen global UHC policy documents sourced from institutions such as WHO, United Nations (UN), and the World Bank (see Additional file 2). These discourses were compared to the dominant discourses identified in the five most recent iterations of the SA NHI policy documents. The scope of this study looked at recent discourse, therefore only policy documents between the years 2010 – 2020 were included in the DA.^6^ The current dominant discourses in global UHC policy documentation reveal discourse of ‘health as a global public good’, and a neoliberal discourse of ‘market-based provision of care’ (Table 3), unpacked below.

**Table 3:** Summary of comparative analytical observations from discourse analysis of global policy documents and SA NHI documents (source: author)

Discourse analysis of global policies on UHC (*n* = 15)	Discourse analysis of SA NHI policies (*n* = 5)
‘Health as a (global) public good’ discourse	
* Underlying theme in texts*: ensuring *all* people can use the health services they need; leaving no one behind; everybody’s business	*Underlying theme in texts*: providing health for all citizens; social solidarity; cross-subsidisation
* Driven by*: principle of universalism which arose at Alma Ata declaration; linked to discourse of health as a human right; global social accountability	*Driven by:* global discourse of universality but conditional for citizens and those who ‘belong’ (nationalist ideology); health as a human right (SA Constitution); government attempting to redress inequalities of the past (political agenda)
Neoliberal discourse	
* Underlying theme in texts:* best way to achieve UHC is through a financing mechanism (health insurance); protecting people from financial hardship; private sectors ingrained role in UHC	*Underlying theme in texts:* NHI Fund as a strategy to move towards UHC; effective and efficient use of resources; private sectors assumed role in NHI
* Driven by*: economic utility; market-based provision of care; assumption of quality care in private	*Driven by:* global discourse; historical context of SA; economic utility; market-based provision of care; scarcity of resources

The discourse of ‘health as a global public good’ was dominant in global texts, linked closely to the principles of universalism and social justice. For example, WHO’s definition of UHC focuses on ensuring *all* people can access the health services they need [38]. The World Bank and WHO’s UHC2030 vision paper is based on the principle of ‘leaving no one behind’ with an explicit commitment to equity and a human-rights based approach [39]. The document places emphasis on health as a social good, making it ‘everybody’s business’ to achieve UHC [39]. This discourse emerged globally at the time of the Alma Ata declaration in 1978 from which the concept of UHC became prominent in response to a rise in global health inequalities and failures to improve health inequities [57]. Since the conception of UHC, “there have occurred substantial shifts in discourse and meaning, shaped by changing international and national contexts and social forces impinging on health systems” [57]. However, the discourse of ‘health as a global public good’ still prevails today.

The current global policy documents also emphasise the principle of social solidarity, and argue that “access to and use of health services enables people to be more productive and active contributors to their families, communities, and society at large” [39]. This is where the ‘global’ comes in, as a globalisation discourse prevailed which assumes it is in the national self-interest of developed countries to invest in the health of poorer countries to the benefit of all [57]. This contributes to the rationale that *everybody* should have access to good quality health care in a global world, which is the underlying assumption of the discourse of ‘health as a global public good’ [57, 58].

The second dominant discourse in the global policy documents was a neoliberal discourse which assumed a market-based model of health care as the right approach in progressing towards UHC. For example, there is an assumption in the policy documents that the best way to achieve UHC is through implementing a health financing reform, specifically health insurance mechanisms [42, 59, 60]. This is linked to the use of the word ‘coverage’ as opposed to ‘care’ in UHC policy which assumes enrolment in an insurance scheme, and suggests the provision of a defined package of care [61].

The global policy documents overwhelmingly assume the role of the private sector in achieving UHC, asserting that “it is now difficult to imagine significant progress on issues of global importance, such as health, food security, sustainable energy and climate change mitigation, without the private sector playing an important role” [38]. The WHO’s definition of UHC is focused on protecting users from *financial* hardship while accessing care [38]. These assumptions in the text have been critiqued to mainstream the role of the marketplace in health, as the “overarching discussion of the current UHC agenda is to make sure that some people can pay for health care, some or a lot of which is provisioned at the marketplace” [58]. This discourse draws from neoliberal ideology which is based on three important assumptions: individualism, free market via privatisation and deregulation, and decentralisation [62].^7^ This encourages competition in provision of health services, but this market-driven model has been noted to fail when it comes to universal provision of health care [63, 64].

Dominant neoliberal environments can promote a UHC model that is market-driven, which through pooling of funds and private provision, can become an “efficient way for private capital to extract profits” [61]. However, UHC discourse also offers a vision of societal transformation with the noble principles of universalism and social solidarity outlined in the ‘health as a global public good’ discourse [58]. Research has highlighted the need for active public participation and involvement in order for this transformation to occur [65]. Although integrated, people-centred care is recognised in global policy documents as being “interdependent and mutually reinforcing” of UHC [66],^8^ there has been criticism of the lack of mainstream operationalisation of these approaches [58].

The SA NHI policy documents reflect dominant discourses similar to the global policies to some extent (Table 3). The most significant difference was that in the SA NHI policy documents, the concept of ‘health as a global public good’ was applied to a country context, so health was seen more as a ‘national’ public good with some instances of quasi-universalism. For example, one of the principles of the NHI Bill is universality defined in the Bill as: “*all* will be able to access the same essential health care benefits regardless of their financial means” [37]. However, in other instances in the Bill, this is clearly restricted to all *citizens*, and there is a concrete list within the Bill of who can access NHI and who must register to do so. This removes the ‘universal all’ that global UHC discourse speaks of, instead replacing it with a list of who will (and by implication those who will not) have access to health care. This is linked to the globalisation discourse of resource scarcity, that there are limited resources which must be protected and provided to those most deserving (SA citizens) [67]. In this case, the concept of universality is softened in the national context, using elements of a globalisation discourse for justification.

Nonetheless, there are still many similarities to the global discourse of ‘health as a global public good’. For example, the principle of social solidarity in the SA NHI policy documents, defined as: “all regardless of their socio-economic status will benefit from a national system of health care, which is based on income cross-subsidies between the affluent and the impoverished and risk cross-subsidies between the healthy and the sick” [37]. This national discourse is influenced by the global discourse with the core value of social justice. This is also driven by the historical context of SA, with the discourse seeking to redress the inequalities of apartheid. Although SA government’s intentions in the policy documents may seem to be to redress the inequalities of the past in SA, NHI has also been criticised as a political tool for the ruling party (the ANC) to garner votes [68].

Dominant neoliberal discourse in the SA NHI policy documents was similar to global UHC discourse. For example, a section on the “socioeconomic benefits” of NHI in the Green Paper focused on how health affects social development and economic productivity, the benefits of which include “having a healthier population, which in turn translates into a productive and effective workforce that grows local businesses, attracts foreign investors and grows the domestic economy” [69]. This narrative carries through all policy documents to the Bill, with much emphasis on the ‘effective’ and ‘efficient’ use of resources. This focus on efficiency is similar to austerity measures seen in the discourse of globalisation, founded from neoliberal economic theory [70]. Much of the NHI Bill is focused on the establishment of an NHI Fund with the aim to, through mandatory prepayment, “achieve universal access to quality health services” [37]. This assumes that a financing mechanism is the way to achieve UHC, based on a market-driven model of health care provision.

Neoliberal discourse is also reflected in the role given to the private sector in the SA NHI policy documents. For example, the Green Paper does not rule out a multi-payer system which implies the possibility of more than one entity financing the health system [69]. In the SA context, this refers to private medical schemes as they are the only alternative to public funding. Although more recent policy iterations clearly state the NHI Fund as the single payer in the system, medical schemes will still exist but be reduced to ‘complementary cover’ (the scope of which is yet to be determined, depending on what the benefits package of NHI will cover, but is not mentioned in the policy documents) [37]. This assumed role of the private sector reflects the neoliberal principle of privatisation of care [62]. It also reflects the historical context of SA where the private sector has a long history and is a powerful socio-political player [22]. The assumption that a market-based model and privatisation of care is the best way to achieve UHC in SA could lead to poor outcomes in the implementation of NHI [62].

The discourse within the SA NHI policy documents is influenced by global UHC discourse, and also by the historical context of SA. Both global and national policy documents reveal somewhat contrasting discourses: the noble principles of universality and social solidarity in the discourse of ‘health as a global public good’ are to some extent incongruous with the discourse of neoliberalism. In the context of SA, these discourses might influence media discourses and public perception, and ultimately affect implementation of NHI. If media discourse is influenced by the discourse of ‘health as a global public good’ and combined with operationalisation of people-centredness and public participation, this could have positive effects on the NHI policy process. If media discourse is overridden by the influence of neoliberal ideology of market-based provision of care, this could have negative implications. However, like the policy documents, one might expect to see these two discourses competing within media representations.

### Content of the SA NHI policy within media

**Fig. 1:**
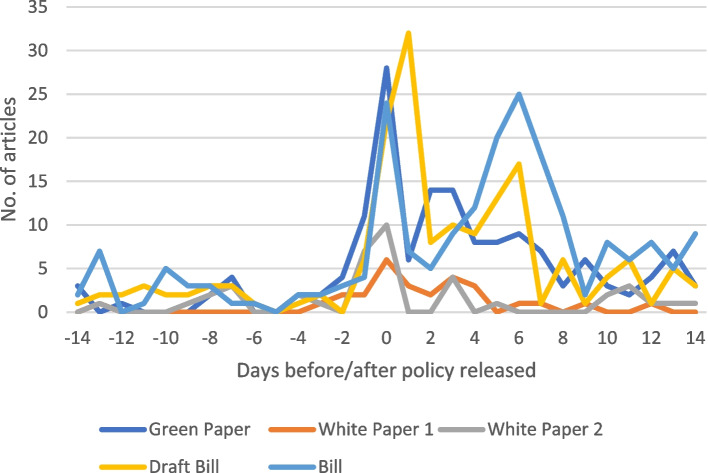
Distribution of media texts published before and after the release of each policy document

This section briefly profiles the content of the media texts collected (*n* = 710) (see Additional file 4 for descriptive statistics of texts collected, and Additional file 5 for a full list of texts collected). Significantly fewer texts were collected for both of the White Papers compared to the other policy documents (Table 4). There was a sharp increase of texts published at each policy document release date, with a relatively increased rate of publications after release (Fig. 1). The Bill and Draft Bill appear to show a second increase of publications beginning four days after the policy release.

**Table 4:** Media texts collected for content analysis

Policy documents	Date released	Dates searched for	No. of articles (with multiples^*^)
Green Paper	2011/08/12	2011/07/29 – 2011/08/26	151 (161)
White Paper 1	2015/12/11	2015/11/27 – 2015/12/25	26 (27)
White Paper 2	2017/06/30	2017/06/16 – 2017/07/14	40 (46)
Draft Bill	2018/06/21	2018/06/07 – 2018/07/05	166 (199)
Bill	2019/08/08	2019/07/25 – 2019/08/22	203 (277)

^*^Texts that were published more than once are included in brackets but were excluded from further analysis

When analysing the ‘tone’ of collected texts towards NHI (Table 5), just less than half (44.6%) of the total texts did not clearly state a position for or against NHI.^9^ A third of total texts (34.7%) were negative in tone, and 21.1% were positive. The number of texts with a negative tone rose considerably at the publication of the Bill in 2019 at which time half (50.9%) of texts were negative in tone. Examples of texts with a positive tone include those that clearly state their position in support of the NHI: “[An NHI] scheme will help protect the poor, provide an enabling environment to prevent further cost escalation in the private insurance market and help secure a wealthier future for South Africans” [71]; and a quote from an interview with MoH, Aaron Motsoaledi: “You need NHI precisely because you want to use this universal health coverage plan to correct the wrong things in the healthcare system” [72]. Examples of texts with a negative tone are: “[the NHI] fails to nail down specifics… [and] is certain to face legal challenges” [73]; and “these gaps in information about the NHI, along with a slew of other problems, indicate that the government’s universal healthcare plans are heading for disaster” [74].

**Table 5:** Tone towards NHI from the media texts collected

	**Green Paper (*n* = 151)**	**White Paper 1 (*n* = 26)**	**White Paper 2 (*n* = 40)**	**Draft Bill (*n* = 166)**	**Bill (*n* = 203)**
Positive	32 (21.2%)	4 (15.4%)	9 (22.5%)	45 (27.1%)	35 (17.2%)
Unclear	61 (40.4%)	18 (69.2%)	19 (47.5%)	56 (33.7%)	65 (32.0%)
Negative	58 (38.4%)	4 (15.4%)	12 (30.0%)	65 (39.2%)	103 (50.7%)

**Fig. 2:**
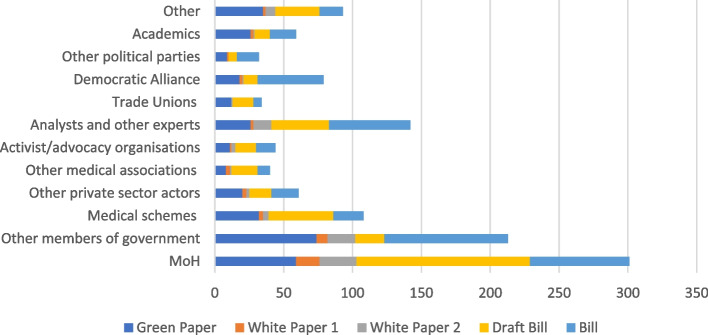
Number of mentions of actors either quoted or published at the release of each policy document

Policy actors were represented in media either through being directly quoted or publishing opinion pieces (Fig. 2). They often expressed a clear position for or against NHI. The key groups of actors represented in media are: the government.^10^ most often the MoH, who strongly supported the implementation of NHI; the private sector, most noticeably medical schemes, who did not directly oppose the principles of NHI but instead advocated their expanded role in its implementation; opposition political parties, primarily the Democratic Alliance, who opposed NHI in its current form; trade unions, who supported the concept of NHI but opposed some aspects of its implementation (such as private sector involvement); and a variety of independent organisations and individuals whose opinions varied based on their own interests. Activist and advocacy organisations such as the People’s Health Movement and Sect. 27 were represented in media as supporting the principles of NHI and health for all. Government actors, including the MoH, featured over 300 times at the release of the two most recent policy iterations. The Democratic Alliance, medical schemes, other private sector actors, and analysts and other experts^11^ were also featured often during this time.

**Fig. 3:**
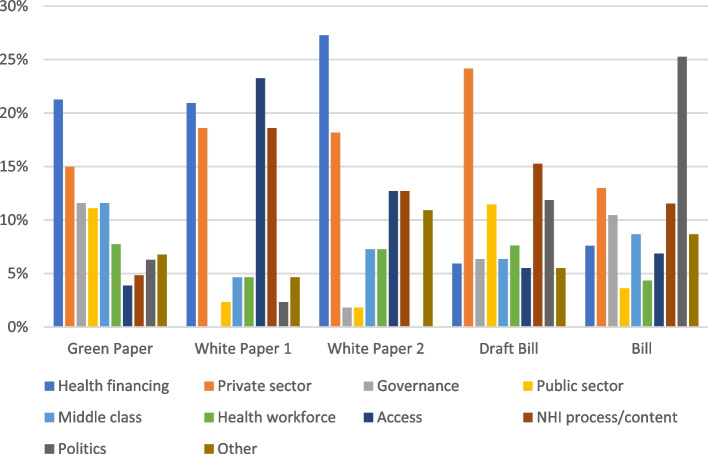
Topics identified in the content analysis distributed by how often they featured (%) at the release of each policy document

Texts focused on a number of different topics. The number of texts focusing on politics (defined as texts which focused on the discussion/contestation between different policy actors in the NHI process) rose considerably at the publication of the two most recent policy documents. No texts focused on politics at the release of the second White Paper, which increased to a quarter of texts at the release of the Bill (Fig. 3). This rise in political contestation is interesting and could explain the second increase in text publications after the release of Draft Bill and Bill as seen in Fig. 1. Other common topics identified in the media texts were (from most to least common): the private sector, health financing, NHI process/content of policy, access, politics, middle class, other, the health workforce, governance and the public sector. Interestingly, health financing was focused on much less at the release of the Draft Bill and Bill.

Unpacking these topics further (Table 6) showed that texts about the private sector were focused on how the private sector might be ‘destroyed’ by NHI, examples include titles such as “*NHI to cut into private care profit*” [75] and “*NHI will kill smaller medical schemes*” [76]. There was a focus on medical schemes who were reduced to offer ‘complementary cover’ to the NHI Fund, which would supposedly lessen their role as they currently provide cover to 16% of the population [25]. Other texts focused on the impact of NHI on middle class taxpayers. ‘Middle class’ is a vague term, especially in a country with such high income inequalities as SA [77]. NHI was seen to be a burden as the middle class would be forced to make mandatory contributions to NHI (e.g. “*NHI contributions will be compulsory”* [78] and *“How you’ll be paying for NHI – more taxes”* [79]).

**Table 6:** List of common topics identified in the content analysis with explanations and examples from the text

**Topic**	**Explanation** (Texts focused on…)	**Examples in media**
Access	Any aspect regarding who can access current system/NHI, including inequalities in the system	Inequality between public and private sectors Access to care regardless of SES Cross-subsidisation Who has access to NHI
Governance	The government and their role/responsibility in NHI	Corruption Bureaucracy Lack of competency
Health financing	Any financing and funding mechanisms of NHI	Unaffordability of NHI Different funding mechanisms How much NHI will cost Removal of medical scheme tax credits More money will not solve the problem
Health workforce	The SA health workforce (public and private)	Shortage of health workforce Doctors leaving the country
Middle class	How NHI will affect ‘you’ (typically implied to be a ‘middle class’ SA citizen)	Middle class ‘paying twice’ Mandatory contributions (no choice) Burden on taxpayers
NHI content/process	Discussion of any of the contents of NHI policy documents or process	Purpose, functions etc. of NHI Pilot projects Release of documents for public comment
Other	All other topics of focus not included on this list	Public participation Clarity needed on many aspects of NHI
Politics	Discussion/contestation between different policy actors in the NHI process (not limited to political parties)	Responses to policy documents (for or against NHI) Questioning constitutionality Government claims/rebuttal to comments
Private sector	Any aspect of the private sector e.g. health facilities, medical schemes	Private sector ‘destroyed’ New role of medical schemes Potential for collaboration with the private sector
Public Sector	Any aspect of the public sector	Fix the quality in the public sector OHSC audits

Key: Office of Health Standards Compliance (OHSC); socio-economic status (SES)

There were some texts focused on who would have access to NHI and some suggested NHI might address current inequalities in the SA health system (e.g. *“Making healthcare equitable”* [80] and *“NHI aims to help everyone”* [81]). These were primarily published by government actors. Content analysis also shows what is missing and texts which focused on health as a public good and people-centred care were not significantly present in media texts published by reporters or other actors other than those in government. This is interesting given the global and national policy discourse of ‘health as a global public good’. In addition, the number of texts with a negative tone towards NHI is interesting to note (Table 5), as global and national policy discourse overwhelmingly assume UHC and NHI to be a ‘good thing’. However, the focus on health financing and the private sector found in media texts might be expected in the context of the neoliberal policy discourse. These findings will be discussed further when unpacking the dominant discourses prevalent in media representations below.

### Dominant discourses within media representations of the SA NHI

The analysis reported above led into a closer analysis of the dominant discourses identified in selected media texts (*n* = 160)^12^ highlighting power and contestation in media representations, with implications for how the SA NHI policy process might unfold. The three most dominant discourses will be unpacked, namely: a neoliberal discourse of ‘privatisation of health care in South Africa’, a biopolitical discourse of ‘class divisions’, and a corruption discourse of the ‘dangers of centralising power’.

#### A neoliberal discourse of ‘privatisation of health care in SA’

The private sector was a common topic of focus within media texts, and further analysis showed that private sector actors in SA are an elite group of actors represented in a particular way in SA media. Neoliberal discourse is used within media to argue for an expanded role for the private sector. This reflects the neoliberal assumption that the success of a reform depends upon privatisation [62]. The neoliberal discourse was also reflected in the overwhelming assumption of quality of care in the private sector which was present in official government communication and reflected in media. At the release of the Green Paper, the then MoH, Aaron Motsoaledi, was quoted by *BBC News* stating: “*the private sector is held up as an example of good service and quality of care*” [82]. However, there is no evidence of a proven higher quality of care in the private sector in SA, therefore this is nothing more than an assumption supporting the neoliberal discourse and market-based provision of care. In addition, this high quality of care was assumed to necessarily come at a high cost. This is an example of how the neoliberal discourse constructs health care as a luxury to be accessed by those who can afford it [83].

The unaffordable cost of private sector care along with the poor quality of care in the public sector were assumed to be the two main issues of the current health system in media texts, labelled as the “terrible twins” [84]. Despite framing of these as the primary issues of the health system, NHI was not a commonly accepted solution amongst actors represented in the texts. Instead, the proposed solution was to increase involvement and collaboration with the private sector. This was endorsed, unsurprisingly, by private sector actors. For example, Ali Hamdulay, the Board of Healthcare Funders (BHF)^13^ chairperson, argued in *The Citizen* that it is *“imperative that the private sector collaborates closely with the department of health and shares their views, insights and knowledge to positively shape the healthcare landscape*” [85].

Another discourse emerged which revealed contestation around the role of medical schemes which was not present at the global or national policy level. This discourse framed NHI as a threat to medical schemes and their members. There was concern over medical schemes future role being reduced to offer “complementary” or “top-up” cover only [86]. Medical schemes, and other private sector actors stated their support for the principles of UHC in media texts, but pushed the narrative that NHI in its current form was not the best way to achieve this. They utilised the lack of clarity around their roles (policy documents were often criticised within media texts for a lack of clarity on a number of issues) to their advantage to support the narrative that medical schemes, and the private sector in general, should play a larger role. This media discourse is similar to both the global and national policy discourse of ‘market-based provision of health care’. This discourse aligns with the interests of private sector actors and makes it easier for them to advocate for an expanded role.

This neoliberal discourse favours the private sector, and private sector voices were regularly highlighted in media. This dominance of the private sector within media is in stark contrast to the lack of representation within media of those whose lives might be most affected by NHI – middle- and lower-income communities and individuals, and other vulnerable groups. Some advocacy organisations were represented within media who argued for greater public participation and inclusion [87, 88], but this narrative was overshadowed by the dominant discourses. There was extremely limited direct representation from vulnerable groups, notably a letter to the editor after the publication of the Green Paper from a group of united informal healthcare workers who were opposed to NHI, stating: *“NHI will mean workers will have to pay an extra 10% tax and then go to a private hospital. So the proposed health plan is just another way to make the private companies and banks that own these private hospitals, richer”* [89].

#### A biopolitical discourse of ‘class divisions’

The second dominant media discourse identified is a biopolitical discourse which shows the reinforcement of social classes within media. Biopolitics is related to the reinforcement of social hierarchy, and the prioritisation of some lives over others [90].^14^ It is assumed firstly that readers of these media texts are primarily middle class taxpayers, and secondly, that they would be negatively affected by NHI. In 2017, at the release of the second White Paper, contestation within media arose over the government statement that medical scheme members would lose their tax benefits in order to pay for NHI. Media texts reported this would have a “devastating effect on the already struggling middle class” [91]. This shows that the middle class is assumed to belong to medical schemes. This framing of a middle class is not the actual statistical majority of the South African population as only 16% of the population belongs to medical schemes, typically in the top two income quintiles [25].

Delineating social classes in this biopolitical discourse is another way to reinforce social hierarchy [92]. SA is no stranger to biopolitics from its apartheid history which utilised race in politics to assert power over certain groups [93]. It has been highlighted again by recent social movements in the country such as the #FeesMustFall movement at SA universities protesting fee increases that primarily excluded Black students from tertiary education [94]. This media discourse similarly suggests the reinforcement of a social hierarchy which prioritises the lives of some over others through social order or class.

The dominant assumption within media was that NHI was an additional burden to an over-stretched middle class. This middle class, similarly to the private sector, were assumed to be victims of NHI. One way that discourse reinforces meaning is through metaphors [83]. The metaphor of the middle class as ‘cash cows’ was utilised within media (e.g. “*You’re the cash cow: Middle class likely to foot the bill for health care*” [95]). A ‘cash cow’ refers to an investment which you can ‘milk’ to yield profits without much maintenance [96]. This implies the middle class would be ‘milked’ to provide funds for NHI, without getting much back in return. This is a market-based metaphor based on individualism and neoliberal ideology. In addition, the primary reason for the supposed outrage of the middle class within media texts was financially-based. For example, that the middle class will now have to “pay twice” – once to contribute to NHI and once for their medical aid. There was an assumption that this would make NHI unfeasible as it would be relying on a small, narrow tax base of middle class taxpayers. There was further outrage within media texts that the contributions to NHI would be mandatory as this would strip individuals of their right to choose. This narrative is rooted in economic liberalism.

In comparison to the focus on the middle class, there is little representation of the rest of the SA population in the media texts. Inequalities in access to health care were highlighted to some extent, mainly supported by direct quotes from official government sources who put forward NHI as a solution to address the inequalities of the health system. They often reiterated the purpose of NHI to increase access to health care for all South Africans and highlighted the principle of social solidarity. Although this narrative appeared in media texts, it never dominated the discourse.

There is a stark contrast here between the media discourse, and the global and national policy discourse which clearly emphasises health for all and the need for social solidarity. What was dominant in the media discourse was the (financial) concern of the supposed middle class (those belonging to medical schemes). This contrasts the lack of discussion in media texts about the majority of South Africans (those who are uninsured) whom NHI might affect quite differently. This comparison of “insured” to “uninsured” again suggests a market-based model of health care provision [83], and prioritises access to health care of some over others. The middle class is assumed to already have access to health care, and will continue to have access to health care within NHI as well. In contrast, there was little mainstream discussion within media about who might be denied access or how inequalities might be perpetuated in the system.

#### A corruption discourse of ‘the dangers of centralising power’

The third dominant discourse identified is a corruption discourse which speaks to ‘the dangers of centralising power’. Governance and politics were common topics of focus within the texts (Fig. 3), which suggests their importance in the NHI policy process. Many media texts casted doubt on the government’s ability to implement NHI successfully. The government’s described history of corruption and poor results were assumed the primary reason for this. A reporter in *Business Day* highlights this: “*this is a government with a dismal track record in implementing many of its own health policies… The state has proved so vulnerable to corruption that investors are rightly worried NHI may do more harm than good”* [97].

Poor governance was assumed to be the reason the public sector quality of care was poor. It was argued that corruption and the public sector needed to be fixed first, separately from NHI implementation, before any other improvement in the system could be achieved. This argument was backed by prominent actors within media such as Alex van den Heever, an economist and academic at Witwatersrand University in SA, and the opposition political party, the Democratic Alliance. The Democratic Alliance shadow MoH, Mike Waters, argued in *Independent Online* that: “*insufficient funding is not the biggest problem facing public healthcare, rather the greatest problems being faced are a lack of accountability, weak and incompetent hospital management, [and] a cumbersome management structure”* [98].

Public sector actors were represented in a negative light within media at different levels of government from hospital managers to the South African Department of Health (SADOH) and MoH [99, 100]. In fact, it was clear that some actors utilised media as a platform to criticise government and the ruling party, the ANC. Throughout the media texts, a slew of government failures, within the health sector and beyond, were listed as to why readers should have little faith in the government implementing another “state-owned enterprise” [74]. Notably, this discourse changed over time. In 2011, allegations of corruption and incompetence featured mainly as opinion pieces and letters to the editor, but over time they became more mainstream with more of a dominant assumption or acceptance that corruption was an issue. Particularly, at the release of the last two policy documents, debates within media on NHI became highly political, with input from a number of different interested parties.

Global UHC policy highlights the importance of good governance, and the need for enhanced transparency and accountability when implementing UHC reforms [38]. The SA NHI policy documents have been described as placing a lot of centralised power in the hands of the SADOH and MoH [37]. Decentralising power is another central principle of neoliberal ideology [62], and persistent claims of corruption in many different countries have often justified the implementation of neoliberal strategies globally [101]. This discourse of corruption within SA media could also be seen as justifying the implementation of a market-based model of health care provision. This corruption discourse is unique to the SA media because of the local context of SA, and many real examples of state capture and corruption in SA continue to be exposed in the health sector and beyond [102, 103]. Whether influenced by local context or broader neoliberal ideology, this corruption discourse, as well as the biopolitical and neoliberal discourses discussed above have implications for the NHI policy process in SA and beyond, discussed below.

## Discussion

Comparison across the various analyses revealed political and ideological contestation of the SA NHI; a similar neoliberal discourse in global and national policy documents, and SA media; a unique discourse of corruption within SA media; and a biopolitical discourse of ‘class divisions’ in contrast with global and national policy discourse. The dominant discourses identified within SA media were influenced by the local context. This section will discuss these findings in turn.

Firstly, the political and ideological contestation of NHI is unique to the SA media discourse. The global policy discourse overwhelmingly assumes that implementing UHC reforms is the ‘right’ thing to do. However, this was not the case in SA media, and other studies in local contexts have also shown political contestation within media relating to UHC reforms [18, 19, 104]. As shown in the results, political contestation within media rose significantly at the publication of the last two policy iterations. The rise in political contestation could be due to a number of reasons and may be linked to the rise of negative texts at the publication of the Bill. One reason could be that as the policy reached final formulation, and the possibility of its implementation became more real, actors may have found their interests to be more at stake. For example, in the Green Paper, a multi-payer system was not ruled out (giving medical schemes increased opportunity in NHI). However, as the process developed, it was clear that the role of medical schemes would be reduced, giving them reason to resist NHI. Another potential reason could be that cases of corruption and state capture in SA were revealed throughout this time [103], decreasing actors’ trust in the government’s ability to implement NHI successfully. In addition, there was much less media attention (substantially less media texts published compared to the other policy documents) at the release of both the White Papers. This change in media coverage is interesting to note. It could be a reflection that the policy at this point was not as contested, or possibly at this time that actors were still trying to influence the policy behind the scenes.

Secondly, a neoliberal discourse is present in both global and national policy documents, and within SA media. There are similar assumptions of a market-based model of provision of health care and privatisation of care. Privatisation is a key neoliberal principle [62]. Voices from the private sector were given space in media texts, but this principle of privatisation went beyond private sector actors being featured in media texts. It was entrenched as a core assumption within the SA media discourse itself, just as both global and national policy discourse accept the role of the private sector without contestation. This neoliberal discourse places emphasis on reforming financing mechanisms, linked to a market-based model of health care provision. Research has shown, however, that moving towards UHC requires more than just addressing the financing of a health system [61].

Thirdly, the biopolitical discourse of social class is in direct contrast to the global and national discourse of ‘health as a global public good’. Although government actors were quoted within media on the importance of social solidarity and improving access to health care, the argument was not strongly present that an important reason to implement NHI was to improve the public good. Discourse of ‘health as a global public good’ was mostly silenced in SA media by neoliberal discourse. The corruption discourse of ‘the dangers of centralising power’ is also rooted in neoliberalism. This discourse is unique to SA media and is influenced by the local context. These findings highlight the difference between global policy discourse, and the reality of health policy processes occurring in local contexts.

There is a dynamic relationship between these more dominant and marginalised discourses, influenced by the local context of SA. What was mostly lacking in the texts was how NHI was a way to progress towards the noble principles of UHC including health equity. This may be a feature of the power and voices represented in media in the SA context such as the powerful role of the private sector. These discourses are located within broader South African discourses on corruption, health, and rights. The national policy of the post-apartheid ruling party, the ANC, has been found to be rooted in neoliberalism [105, 106]. Corruption is highly prevalent in the country, and a governance crisis exists, where leaders have exploited the vulnerability of citizens rather than utilising the state’s power to promote the public interest [107, 108]. It would be useful to investigate this corruption discourse further, as well as to explore how other health related and broader discourses shape the NHI discourse.

There are several ways representations within SA media might influence the SA NHI policy process (Table 7). Firstly, SA media discourses reinforce privatisation in provision of health care. As privatisation of care is a core principle of neoliberalism [62], there is no surprise that private sector actors will benefit from the dominating neoliberal discourse prevalent in SA media. In SA, the private sector is largely unregulated and has operated on motives that have excluded the majority of the population and perpetuated inequality for over 25 years [28]. When thinking about powerful actors, such as the private sector, in health policy processes, it is important to understand what their interests are and what they might have to gain or lose from the process [13]. For example, private hospitals and hospital groups featured less in media. One explanation for this might be that their role was less threatened as private hospitals and providers can be accredited and contracted through NHI, and ultimately still gain profit. Medical schemes were more often featured in media as their role appeared more at threat, reduced to ‘complementary cover’. Globally, there have been examples where rampant, poorly regulated private sectors have caused damage to health systems and the health of populations [109, 110]. This should be heeded in the context of SA. For example, the SA Health Market Inquiry (HMI) report released in 2019 (the most comprehensive investigation carried out on the private sector in SA) provided wide-ranging recommendations to regulate the private sector which have been described as critical for the success of NHI [111].

**Table 7:** Top ten key take-away messages

**Discourses within SA media relating to the SA NHI reveal:**
1. Political and ideological contestation in the debate about NHI
2. A neoliberal discourse which assumes privatisation as a key aspect of successful NHI implementation
3. A biopolitical discourse which reinforces a social hierarchy in SA in which some groups are prioritised over others
4. A corruption discourse of ‘the dangers of centralisation’ unique to SA media
From these discourses, the following conclusions can be drawn:
5. Neoliberal discourses within SA media e.g., on privatisation are similar to global and national policy discourse, but the other dominant discourses within SA media are unique to local context
6. Representations within media reinforce inequalities in the SA health system, with the potential to influence the implementation of an NHI with continued unequal access to health care
7. Media representations perpetuate a lack of trust in government which can negatively impact access to health care
8. The discourses in this particular media do not represent the lived reality of the majority of South Africans and hinder public participation in the policy process
9. There are differences between global policy discourse and how health policy processes unfold in a local context, this should be considered when implementing UHC reforms in other LMICs
10. Further studies are required to investigate other diverse media formats and languages; explore media’s role in health policy processes in other contexts; and investigate how to utilise media within health policy processes to enhance health outcomes and access to health care

Secondly, representations within SA media reinforce social hierarchy which may have negative repercussions on access to health care. SA media discourse is concerned with the economic elite and largely ignores the broader social questions of justice and equality. This has been found in previous research highlighting the lack of diversity in SA media [48]. In some ways, this might be expected when analysing English-only print and online media in SA, as the elite in SA are their assumed target market [112]. SA media discourses prioritise some individuals and groups over others. SA has a history of oppression and social inequality [20]. This reinforcement of social hierarchy within media may influence public perceptions to perpetuate this social divide. The current two-tier health system in SA provides different levels of access to different members of the population [28]. There is risk for this reinforcement of social division to influence decision-making by policy actors (particularly those developing formal policy and influencing how policy is translated into practice) towards implementing an NHI in another two-tiered form, continuing to prioritise some individuals’ access to health care over others. As Prince [65] explains, “simply creating a new financing bureaucracy does not guarantee equity and universal access in a health system, because it does not tackle social fractures between people or change relationships and identities, the social part of solidarity”.

Thirdly, media representations perpetuate a lack of trust in government which can negatively impact access to health care. The discourse of corruption within SA media reinforces a lack of trust in the public health sector. Trust is important in a health system, and it has been established that health system performance is associated with trust in governance [113]. It has also been found that trust in government is lower in countries where the health system is financed to a greater degree by private sources [114]. Given SA’s large private sector, it would then be unsurprising that trust in SA government is low. SA is a country with low social cohesion, and the values of solidarity and collective action do not function at a national level [115, 116]. Negative representations of NHI can lessen public trust, which in turn can decrease access to the health system [10, 117].

Finally, representations within SA media hinder public participation in the NHI policy process. There is lack of direct representation of the general public’s voices in SA media. A study in Uganda analysing media discourse around abortion, a controversial health policy issue, highlighted the significance of the exclusion of certain statements or actors [118]. What is missing in SA media is representation from the middle- and lower-income groups – those whom NHI is meant to affect most. A study conducted in 2014 to explore the SA public’s perspectives of NHI highlighted discrepancies between the intended health policy reform and the lived reality of public health care consumers [119]. Similarly, the dominant discourses within SA media ignore the lived reality of most South Africans and reinforce a lack of public participation in the policy process. There is also a lack of public participation and accountability in NHI policy, for example there is a lack of direct representation from the public on committee structures [120]. Public participation has been described as necessary to implement real change away from neoliberal ideology [65], but has been limited in the context of the SA NHI policy. Public awareness of the NHI is low [46, 121], with exclusion of vulnerable groups such as migrants and youth in the policy process [122, 123].

Through understanding these findings, and the implications they may have on the policy process, several recommendations can be made in the context of SA and other LMIC settings (see Table 8 for specific recommendations for policy actors). The first is that the SA NHI policy process requires more genuine public participation. Global policy documents highlight the importance of public participation in moving towards UHC [66]. To change the current ‘social contract’ requires active public participation and placing people, communities, and their wellbeing at the centre of the discussion [58, 65]. Pandey [58] suggests that structural and organisational reconfiguration is required to enable people and communities to become producers of health care as opposed to mere consumers. SA media could be utilised in this process by providing a platform to make individual and community voices heard, particularly those most vulnerable in society, instead of reproducing discourses that benefit an elite few. This has been achieved successfully in other settings through media advocacy by a range of grassroots activists, public health groups, social advocates, and researchers [124–127]. Media advocacy links public health and social problems to inequities in social arrangements, and has the primary goal of reducing power imbalance [124].

**Table 8:** Recommendations for NHI policy actors in SA

• SA policymakers should be aware of how media discourses prioritise the interests of the private sector
• Policymakers should collaborate with media actors to investigate how to minimize harmful representations in mainstream media; and make media more inclusive with the goal to enhance health outcomes
• Policymakers and media actors should work together with researchers to explore alternative media formats to harness their potential successes
• Capacity building of all policy actors is required to increase reflexivity, empowering them to think critically about the discourses they are reproducing with the goal to increase inclusion of vulnerable groups and communities, and build trust and social cohesion to move towards UHC
• All interest groups, particularly civil society and private sector actors, should be engaged throughout this process

This study provided an overall analysis of how the SA NHI policy was represented in mainstream media, with implications for how this might influence the policy process, and provision of specific recommendations for NHI policy actors. Given the exploratory nature of this study, and its broad scope, these results generate multiple future research agendas which can be explored. Recommendations for further study include investigation of the dynamic relationships between actors, and how these shape discourse. The way media discourse impacts on the NHI debate could be explored. Although this study looked at discourse over an extended period of time, further insight into how the discourse evolved, and its influences and impacts can be investigated. It should be recognised that media is not a homogenous block, and actors can use media to set discourses in line with their own interests. Different media ownerships and the influence of ownership on discourse should be investigated. Analysing the process of how global discourses become contextualised in SA and the dynamic relationships between actors, context, and policy processes could be further explored. Furthermore, the role that COVID-19 and related discourses have on NHI discourse will be a relevant area for investigation. Mainstream media representations of the pandemic influenced global discourses and the spreading of disinformation about COVID-19. Several lessons can be learnt from the influence of the pandemic on the SA health system which could inform NHI and should be investigated further [128].

Other media formats and languages should be investigated further in SA and other local contexts. Print and online texts were selected for this study as they are the most widely read in SA, and allow an in-depth focus on a specific media format [10, 129]. However, given the diversity in SA, and that print and online English media target only a specific group [48], it cannot be concluded that all forms of media reproduce similar discourses. While in this study media representations appear to be reinforcing hierarchy and social dominance, other media formats may reveal different findings. It is crucial for researchers to explore more diverse languages and forms of media to understand other representations and dominant discourses before drawing conclusions more broadly in other contexts.

## Conclusion

Media not only represents discourse, but shapes the discourse around us [2]. Discourse, as defined earlier, is relatively lasting ways of representing particular social processes which tend to *focus* on some aspects and *marginalise* others [6]. Discourse within SA media *focuses* on the effect of NHI on elite and powerful actors (and their effect on NHI). It *marginalises* everyday citizens, and the greater questions of access and inequality in health. SA media discourses are clearly influenced by local context, and in some instances contrasted the global and national policy discourse. The neoliberal discourse dominating SA media reinforces social, economic and political divides.

Although discourse of ‘health as a global public good’ contains noble principles such as universalism and social solidarity, this discourse can be influenced through a dominant neoliberal environment for private capital to extract profits and continue to perpetuate inequalities in access to health care [58, 61]. The representations within SA media may contribute to future implementation of an NHI policy out of touch with the lived reality of everyday citizens, perpetuating inequalities in access to health care. However, media has the potential to change this and move towards social transformation [124, 130, 131]. Research should investigate how to utilise media within health policy processes to work towards decreasing social and health inequalities.

Media has the potential to be utilised as a powerful tool to further progressive policy agendas. There is an urgent need for policy actors, including policymakers, civil society, and media actors, to build sustainable and collaborative networks in local contexts to explore ways to construct media environments which re-produce discourses that influence health policy processes in positive ways. The ongoing rise of new and innovative forms of media such as social media bring a window of opportunity for this change. There is an opportunity to research and formulate innovative ways to utilise media as part of health policy processes and health systems to move away from neoliberal dominance and build public trust and social cohesion, towards an equitable and just society for all.

*“We need to help put into the public space of language a role for human beings that is not merely based in market productivity but in having a socially meaningful and morally coherent life and death”*

-- Malone [83]

## Supplementary Information

**Additional file 1.** Summary of contextual factors and policy content of SA NHI policy documents

**Additional file 2.** Global UHC policy documents and substantiating key literature

**Additional file 3. List of public submissions to the SA NHI policy documents **

**Additional file 4.** Descriptive statistics of media texts collected

**Additional file 5.** List of primary print and online media texts

## Data Availability

The datasets used and/or analysed during the current study are available from the corresponding author on reasonable request.

## Abbreviations

ANC: African National Congress
BHF: Board of Healthcare Funders
DA: Discourse analysis
GDP: Gross Domestic Product
HIC: High-income countries
HMI: Health Market Inquiry
HPSR: Health Policy and Systems Research
LMIC: Low- and middle-income countries
MoH: Minister of Health
NGO: Non-governmental organisation
NHI: National Health Insurance
OHSC: Office of Health Standards Compliance
PHM: People’s Health Movement
SA: South Africa
SADOH: South African Department of Health
SAPA: South African Press Associations
SES: Socio-economic status
UHC: Universal health coverage
UN: United Nations
WHO: World Health Organisation

## Footnotes

1 For the purpose of this study, unless specifically stated, ‘media’ refers to mainstream media defined as traditional and established forms of mass news media (including broadcast, print, and digital formats) that aim to reach a large audience [132]. It is recognised that other diverse forms of media exist, such as social media platforms, and their role in health policy processes should also be investigated.

2 Representation is a process in which members of a culture use language to produce meaning [133].

3 For this study, the ‘private sector’ will exclude private health providers and facilities which do not operate on a for-profit basis and traditional health practitioners as they have histories in SA distinct from the rest of the private sector. Not-for-profit private providers in SA are mainly non-governmental organisations (NGOs) which provide services at a primary care level and mostly funded through philanthropy [134]. Traditional health practitioners evolved differently in SA, and have historically been excluded from the formal health system [135]. The representation of these actors in media might be different and should be explored further.

4 The five most recent SA NHI policy documents are: the 2011 Green Paper, the 2015 White Paper, the 2017 White Paper, the 2018 Draft Bill and the 2019 Bill.

5 Only texts with a main focus on NHI were selected. Texts that only mentioned NHI, or focused primarily on other topics were excluded.

6 As discourse is continuously evolving and shaped by historical context [5], important policy documents outside of this time were briefly analysed to provide context to the current discourse, but DA was not conducted on these documents. Key literature identified in the scoping review on global UHC discourse, and public submissions to the SA NHI policy documents were utilised to substantiate DA of the policy documents. These are listed in Additional file 2 and Additional file 3 respectively.

7 A neoliberal economic doctrine was adopted internationally by institutions, donors and developing countries in the 1980’s and advocated liberalisation of trade, finance and monetary systems, and reduced government involvement [57].

8 People-centred health services are an approach to care which sees individuals, families, and communities as participants in and beneficiaries of health systems. This requires a trusted health system that adopts the perspectives of these people, and responds to their needs and preferences [66].

9 Tone was assessed through clear assertions in media texts stating support of or opposition to NHI.

10 A neoliberal economic doctrine was adopted internationally by institutions, donors and developing countries in the 1980’s and advocated liberalisation of trade, finance and monetary systems, and reduced government involvement [57].

11 Independent organisations, or individuals (experts/professionals) who provided analysis/opinions on NHI within media texts including legal firms, think tanks, economists, and financial advisers.

12 Independent organisations, or individuals (experts/professionals) who provided analysis/opinions on NHI within media texts including legal firms, think tanks, economists, and financial advisers.

13 The BHF is the representative organisation of 95% of private medical schemes throughout South Africa [136].

14 Biopolitics and biopower are terms developed by Foucault [137] to “ensure, sustain, and multiply life”, essentially to give order to life, and denote social and political power over life.
