# Supplementary material for: A discourse and content analysis of representation in the mainstream media of the South African National Health Insurance policy from 2011 to 2019
Source: BMC Public Health. 2023 Feb 7;23:279. doi: 10.1186/s12889-023-15144-6 (PMC9904875; doi:10.1186/s12889-023-15144-6)
Supplement: Supplementary file 1 — Additional file 1. Summary of contextual factors and policy content of SA NHI policy documents [file 12889_2023_15144_MOESM1_ESM.docx]

**Additional File 1:** Summary of contextual factors and policy content of SA NHI policy documents

|  | **Date Released** | **Contextual factors** | **Policy Content** |
| --- | --- | --- | --- |
| Green Paper | 12 August 2011 | MoH: Aaron Motsoaledi;  Human Resources for Health strategy released  Corruption, growing social and economic inequalities and weakening state institutions | More a philosophical approach to ensuring universal access with a focus on PHC; statements of intent but form and implementation unclear.  Emphasis on “Re-engineering PHC” with practical strategies to implement this.  Creation of a single fund with accreditation of public and private providers.  Limited access to ‘South Africans and legal permanent residents’; refugees and asylum seekers covered in line with Refugee Act but not clear what they are entitled to.  Suggests shift of (financial) resources from private to public.  Silent on form of taxation to finance NHI, co-payments discouraged but not excluded.  Building of flagship academic hospitals with public-private partnerships. Multi-payer system mentioned; public still have “real choice” about whether to have additional medical aid.  NHI fund will be autonomous government-owned entity with CEO accountable to MoH.  District health system to deliver services |
| White Paper 1 | 10 December 2015 | MoH: Aaron Motsoaledi  Anticipated release since early 2012 – many delays  Policy process took place behind closed doors: Ministerial-appointed cabinet committee bound by confidentiality  Poor reporting and feedback from NHI pilot districts  Then President Jacob Zuma’s corruption and other state capture begins to be exposed  Mmusi Maimane succeeds Helen Zille as the Leader of the Democratic Alliance, the opposition party  #FeesMustFall protest begins in October  Xenophobic attacks increase in SA | Policy repeats importance of a high quality, equitable and publicly driven health system but certain processes described are in contradiction with these goals.  PHC the “heartbeat of the NHI” with some practical, concrete strategies to implement this  NHI fund will be a single fund with accredited, contracted, and certified public and private providers  South African and permanent residents have access; refugees covered in terms of Refugee Act; asylum seekers can access emergency services; all others need to pay.  Five financing options proposed with mix of VAT, surcharge on income tax and payroll tax; co-payments explicitly abolished.  Medical Schemes Act will only be amended once NHI fully implemented to provide complementary cover  Lack of delineation of powers and functioning of the NHI Fund and NHI commission. |
| White Paper 2 | 30 June 2017 | MoH: Aaron Motsoaledi  Life Esidemeni tragedy exposed (death of 143 patients at psychiatric facilities from causes of starvation and neglect)  Then President Jacob Zuma faces charges of corruption, money-laundering and racketeering  Further examples of state capture and scandal exposed | Little change from White Paper two years previously.  Focus on PHC with practical strategies.  Single fund with accreditation and contracting through public and private providers.  South Africans and permanent residents entitled to access; refugees, asylum seekers and irregular migrants covered in terms of the Refugees Act.  Five financing scenarios proposed with increased VAT, surcharge on income tax, and payroll tax; co-payments abolished.  Medical schemes continue to play a role, will eventually offer complementary cover.  Acknowledges corruption/lack of accountability but no clear implementation strategies to prevent it. |
| Draft Bill | 21 June 2018 | MoH: Aaron Motsoaledi  Released with Medical Schemes Amendment Bill  Cyril Ramaphosa becomes President in February following Jacob Zuma’s resignation  Former President Jacob Zuma appears in court three times | Significant change from White Paper, primarily legislative and highly concentrated on financial aspects.  Confuses PHC approach with primary level of care.  Accredited and contracted public and private providers.  South Africans and permanent residents entitled to access; refugees who have been granted asylum and those awaiting decision on their asylum application are entitled to only a selected set of services; for undocumented migrants, there is no entitlement.  Sources of funding are vague and ambiguous  Complementary, voluntary health insurance still leaves room for medical schemes.  Power concentrated in hands of Board and MoH. |
| Bill | 8 August 2019 | MoH: Zweli Mkhize  Former President Jacob Zuma appeared at the Judicial Commission of Inquiry into Allegations of State Capture; criticism that current administration has not dealt decisively with corruption and state capture | Primarily formal and legislative but written without context.  Focus on health financing reform creating a single fund to contract with accredited public and private providers.  South Africans and permanent residents are entitled to access; exclusion of asylum seekers and undocumented migrants.  Vagueness on funding options.  Medical schemes to offer complementary cover once NHI is fully implemented.  Power heavily centralised to the MoH. |

**References**

1. SADOH. National Health Insurance in South Africa: policy paper. 2011. Available here: <http://www.greengazette.co.za/notices/national-health-act-no-61-of-2003-policy-on-national-health-insurancedraft_20110812-GGN-34523-00657>. Accessed 30 January 2020.

2. SADOH. National Health Insurance for South Africa: towards universal health coverage. 2015. Available here: <https://www.health-e.org.za/wp-content/uploads/2015/12/National-Health-Insurancefor-South-Africa-White-Paper.pdf>. Accessed 28 January 2020.

3. SADOH. National Health Insurance policy: towards universal health coverage. 2017. Available here: <https://www.gov.za/sites/default/files/gcis_document/201707/40955gon627.pdf>. Accessed 28 January 2020.

4. SADOH. National Health Insurance Bill draft. 2018. Available here: <https://www.gov.za/sites/default/files/gcis_document/201806/41725gon635s.pdf>. Accessed 1 February 2020.

5. RSA. National Health Insurance Bill [B11-2019]. 2019. Available here: <https://www.gov.za/sites/default/files/gcis_document/201908/national-health-insurance-bill-b-11-2019.pdf>. Accessed 22 January 2020.

6. Parliamentary Monitoring Group. NHI timeline: key dates and events. 2019. Available here: <https://pmg.org.za/blog/NHI%20Timeline:%20Key%20dates%20and%20events> Accessed 7 March 2020.
