# Supplementary material for: A discourse and content analysis of representation in the mainstream media of the South African National Health Insurance policy from 2011 to 2019
Source: BMC Public Health. 2023 Feb 7;23:279. doi: 10.1186/s12889-023-15144-6 (PMC9904875; doi:10.1186/s12889-023-15144-6)
Supplement: Supplementary file 2 — Additional file 2. Global UHC policy documents and substantiating key literature [file 12889_2023_15144_MOESM2_ESM.docx]

**Additional file 2**: Global UHC policy documents and substantiating key literature

**Table A** Global UHC policy documents analysed

| **Author (Journal) or Institution** | **Year** | **Title** |
| --- | --- | --- |
| Kutzin J (Bulletin of the WHO) | 2013 | Health financing for universal coverage and health system performance: concepts and implications for policy |
| UN | 2013 | United Nations general assembly resolution 67/81 |
| UN | 2019 | Political declaration of the high-level meeting on universal health coverage |
| WHO | 2010 | World health report 2010: health systems financing: the path to universal coverage |
| WHO | 2013 | World health report 2013: research for universal health coverage |
| WHO | 2014 | Making fair choices on the path to universal health coverage: final report of the WHO consultative group on equity and universal health coverage |
| WHO | 2015 | Health in 2015: from MDGs to SDGs |
| WHO | 2015 | WHO global strategy on people-centred and integrated health services |
| WHO | 2019 | Primary health care on the road to universal health coverage |
| WHO & World Bank | 2017 | Healthy systems for universal health coverage – a joint vision for healthy lives |
| WHO & World Bank | 2017 | Tracking universal health coverage: 2017 Global monitoring report |
| WHO & World Bank | 2019 | Global monitoring report on financial protection in health 2019 |
| World Bank | 2013 | The impact of universal coverage schemes in the developing world: a review of the existing evidence |
| World Bank | 2015 | Going Universal: How 24 countries are implementing universal health coverage reforms from the bottom up |
| World Bank | 2019 | High-performance health financing for universal health coverage |

**Table B** Relevant literature from the scoping review utilised to substantiate the analysis of the global policy documents

| **Author (Journal) or Institution** | **Year** | **Title** |
| --- | --- | --- |
| Abadía‐Barrero CE (Medical Anthropology Quarterly) | 2016 | Neoliberal justice and the transformation of the moral: the privatization of the right to health care in Colombia |
| Arrow JK (American Economic Review) | 1963 | Uncertainty and the Welfare Economics of Medical Care |
| Bisht R (Indian Journal of Public Health) | 2013 | Universal health care: the changing international discourse |
| Cueto M (American Journal of Public Health) | 2004 | The origins of primary health care and selective primary health care |
| People’s Health Movement | 2014 | The current discourse on universal health coverage (UHC) |
| Prince RJ (Michael) | 2017 | Universal Health Coverage in the Global South: New models of healthcare and their implications for citizenship, solidarity and the public good |
| Horton R, Clark S (The Lancet) | 2016 | The perils and possibilities of the private sector |
| Lagomarsino G, Garabrant A, Adyas A, et al. (The Lancet) | 2012 | Moving towards universal health coverage: health insurance reforms in nine developing countries in Africa and Asia |
| Mackintosh M, Channon A, Karan A, et al. (The Lancet) | 2016 | What is the private sector? Understanding private provision in the health systems of low-income and middle-income countries |
| McGregor S (International Journal of Consumer Studies) | 2001 | Neoliberalism and health care |
| Mishra A (Social Medicine) | 2016 | Unpacking universal health coverage in India: Implications for health |
| Naidoo S (Journal of Public Health) | 2012 | The South African national health insurance: a revolution in health-care delivery! |
| Pandey K (Globalisation and Health) | 2018 | From health for all to universal health coverage: Alma Ata is still relevant |
| Stuckler D, Feigl A, Basu S (Background paper for the global symposium on Health Systems Research | 2010 | The political economy of universal health coverage |
