# Supplementary material for: A discourse and content analysis of representation in the mainstream media of the South African National Health Insurance policy from 2011 to 2019
Source: BMC Public Health. 2023 Feb 7;23:279. doi: 10.1186/s12889-023-15144-6 (PMC9904875; doi:10.1186/s12889-023-15144-6)
Supplement: Supplementary file 3 — Additional file 3. List of public submissions to the SA NHI policy documents [file 12889_2023_15144_MOESM3_ESM.docx]

**Additional file 3:** List of public submissions to the SA NHI policy documents^[[1]](#footnote-1)^

| **Organisation/Institution/**  **Author** | **Title** |
| --- | --- |
| *Green Paper 2011* | |
| Council for Medical Schemes | Comments on the National Health Insurance Policy Paper of 12 August 2011 |
| Discovery Health | Discovery Health (Pty) Ltd and Discovery Health Medical Scheme comments on Government Gazette No. 34523, on the National Health Act, 2003. Green Paper: Policy on National Health Insurance |
| Helen Suzman Foundation | Submission to National Department of Health: National Health Insurance: Green Paper |
| PathCare | Written Submission on the Policy Paper: National Health Insurance in South Africa |
| People’s Health Movement | The Green Paper on National Health Insurance: Civil society discussion document |
| Rural Doctors Association of South Africa | Submission on the Green Paper on National Health Insurance |
| South African Medical Association | Submission of comments on National Health Insurance (NHI) Green Paper |
| South African Private Practitioners’ Forum | SAPPF submissions on the Green Paper on National Health Insurance |
| Van den Heever AM | Evaluation of the Green Paper on National Health Insurance |
| *White Papers 2015/2017* | |
| AfriBusiness | Comment on the NHI White Paper as published in the Government Gazette 39506, Notice No. 1230 on 11 December 2015 |
| Free Market Foundation | Submission to the National Department of Health: National Health Insurance White Paper |
| Hospital Association of South Africa | HASA NHI White Paper submission |
| Helen Suzman Foundation | Submission to the National Department of Health: The White Paper on National Health Insurance for South Africa |
| Life Healthcare Group | Life Healthcare Group’s White Paper response to the National Health Insurance plan |
| London L | Comments on the White Paper on a National Health Insurance for South Africa: Towards Universal Health Coverage: Report to the Foundation for Human Right (FHR) |
| People’s Health Movement | Comment on the National Health Insurance White Paper: Submission by the People's Health Movement of South Africa |
| People’s Health Movement | Young People's recommendations on South Africa's NHI White Paper |
| PPO Serve | PPO Serve comments on the NHI White Paper |
| Rural Doctors Association of South Africa | Submission on National Health Insurance White Paper. |
| Section27 | Section27 submission on the NHI White Paper. |
| South African Institute of Race Relations | Submission to the Department of Health Regarding the White Paper on National Health Insurance for South Africa |
| South African Medical Association | Submission to Minister of Health, National Department of Health: Comments in respect of White Paper for National Health Insurance for South Africa towards Universal Health Coverage |
| South African Private Practitioners’ Forum | South African Private Practitioners Forum response to NHI White Paper |
| *Draft Bill 2018* | |
| Helen Suzman Foundation | Submission to the Department of Health on the National Health Insurance Bill 2018 and the Medical Schemes Amendment Bill 2018 |
| People’s Health Movement | People's Health Movement submission on National Health Insurance Bill |
| Section27 & Treatment Action Campaign | Section27 and Treatment Action Campaign submission: Draft National Health Insurance Bill, 2018, Draft Medical Schemes Amendment Bill, 2018, Draft National Quality Improvement Plan |
| South African Institute of Race Relations | Submission to the Department of Health regarding the National Health Insurance Bill of 2018 and the Medical Schemes Amendent Bill of 2018 |
| *Bill 2019* | |
| Collaboration for Health Systems Analysis and Innovation | Collaboration for Health Systems Analysis and Innovation (CHESAI) submission to Parliament on the National Health Insurance Bill 2019 |
| Financial Intermediaries Association of South Africa | Comments on National Health Insurance Bill |
| HealthMan | Commentary by HealthMan to the Portfolio Committee on Health  National Parliament of the RSA on Bill 11 of 2019: Proposed National Health Insurance Act |
| People’s Health Movement | The People’s Health Movement of South Africa Submission on the National Health Insurance Bill |
| Public Health Association of South Africa | Written submission to the parliament portfolio committee on health, on the National Health Insurance Bill [B11 - 2019] |
| South African Medical Association | The South African Medical Association submission to: The Parliamentary Portfolio Committee on Health in respect of: The National Health Insurance Bill |
| South African Private Practitioners’ Forum | Submission on NHI Bill to the Portfolio Committee on Health |
| Section27 & Treatment Action Campaign | Submission on the National Health Insurance Bill 2019 |
| University of Cape Town | Towards Universal Health Coverage: Responses to the 2019 National Health Insurance Bill to the Portfolio Committee on Health, Parliament of South Africa |
| University of Stellenbosch | Submission by the Faculty of Medicine and Health Sciences Stellenbosch University on the National Health Insurance Bill |
| Van den Heever AM | National Health Insurance Policy Bill review |

1. A list of public submissions was compiled from the website: www.nhisa.co.za, and further public submissions were identified and included for analysis via Google [↑](#footnote-ref-1)
