# Supplementary material for: A discourse and content analysis of representation in the mainstream media of the South African National Health Insurance policy from 2011 to 2019
Source: BMC Public Health. 2023 Feb 7;23:279. doi: 10.1186/s12889-023-15144-6 (PMC9904875; doi:10.1186/s12889-023-15144-6)
Supplement: Supplementary file 4 — Additional file 4. Descriptive statistics of media texts collected [file 12889_2023_15144_MOESM4_ESM.docx]

**Additional file 4:** Descriptive statistics of media texts collected

|  | **Green Paper**  **(*n* = 151)** | **White Paper 1**  **(*n* = 26)** | **White Paper 2**  **(*n* = 40)** | **Draft Bill**  **(*n* = 166)** | **Bill**  **(*n* = 203)** |
| --- | --- | --- | --- | --- | --- |
| **Author** |  |  |  |  |  |
| **News reporter** | 67 (44.4%) | 19 (73.1%) | 19 (47.5%) | 102 (61.4%) | 117 (57.6%) |
| **News reporter, unnamed** | 21 (13.9%) | 1 (3.9%) | 6 (15.0%) | 27 (16.3%) | 30 (14.8%) |
| **Editor** | 16 (10.6%) | 1 (3.9%) | 2 (5.0%) | 5 (3.0%) | 13 (6.4%) |
| **News agency** | 14 (9.3%) | 2 (7.7%) | 6 (15.0%) | 3 (1.8%) | 10 (4.9%) |
| **Government** | 3 (2.0%) | 1 (3.9%) | 1 (2.5%) | 3 (1.8%) | 5 (2.5%) |
| **Academic** | 9 (6.0%) | 1 (3.9%) | 1 (2.5%) | 3 (1.8%) | 9 (4.4%) |
| **Private organisations** | 7 (4.6%) | 0 (0.0%) | 3 (7.5%) | 6 (3.6%) | 4 (2.0%) |
| **Other: individuals** | 13 (8.6%) | 1 (3.9%) | 0 (0.0%) | 6 (3.6%) | 6 (3.0%) |
| **Other: organisations** | 1 (0.7%) | 0 (0.0%) | 2 (5.0%) | 11 (6.6%) | 9 (4.4%) |
| **Publisher** |  |  |  |  |  |
| **IOL** | 55 (36.4%) | 4 (15.4%) | 6 (15.0%) | 46 (27.7%) | 56 (33.7%) |
| **Media24** | 26 (17.2%) | 8 (30.8%) | 11 (27.5%) | 29 (17.5%) | 32 (19.3%) |
| **Times** | 47 (31.1%) | 4 (15.4%) | 6 (15.0%) | 31 (18.7%) | 36 (21.7%) |
| **Other** | 23 (15.2%) | 10 (38.5%) | 17 (42.5%) | 60 (36.1%) | 79 (47.6%) |
| **Word count** |  |  |  |  |  |
| **1 – 250** | 21 (13.9%) | 5 (19.2%) | 10 (25.0%) | 23 (13.9%) | 22 (10.8%) |
| **251 - 500** | 54 (35.8%) | 9 (34.6%) | 14 (35.0%) | 50 (30.1%) | 61 (30.0%) |
| **501 - 1000** | 64 (42.4%) | 8 (30.8%) | 11 (27.5%) | 68 (41.0%) | 93 (45.8%) |
| **1001+** | 12 (7.9%) | 4 (15.4%) | 5 (12.5%) | 25 (15.1%) | 27 (13.3%) |
| **Type of media** |  |  |  |  |  |
| **Report** | 99 (65.6%) | 22 (84.6%) | 29 (72.5%) | 129 (75.9%) | 153 (75.4%) |
| **Editorial** | 16 (10.6%) | 1 (3.9%) | 2 (5.0%) | 5 (3.0%) | 11 (5.4%) |
| **Opinion piece** | 22 (14.6%) | 3 (11.5%) | 8 (20.0%) | 28 (16.9%) | 39 (19.2%) |
| **Other** | 14 (9.3%) | 0 (0.0%) | 1 (2.5%) | 4 (2.4%) | 0 (0.0%) |
