# Supplementary material for: A discourse and content analysis of representation in the mainstream media of the South African National Health Insurance policy from 2011 to 2019
Source: BMC Public Health. 2023 Feb 7;23:279. doi: 10.1186/s12889-023-15144-6 (PMC9904875; doi:10.1186/s12889-023-15144-6)
Supplement: Supplementary file 5 — Additional file 5. List of primary print and online media texts [file 12889_2023_15144_MOESM5_ESM.docx]

**Additional file 5:** List of primary print and online media texts

| **Date** | **Title** | **Publication** | **Source** |
| --- | --- | --- | --- |
| 2011/07/29 | Sickening costs | The Star | SAM |
| 2011/07/29 | Healthy engagement | Financial Mail | PressReader |
| 2011/07/29 | Cost of quality | Financial Mail | PressReader |
| 2011/07/31 | Anti-collusion legislation blocks moves to overhaul health care | Sunday Independent/ Sunday Tribune | SAM |
| 2011/08/04 | Public and private sectors have a role to play in the National Health Insurance (NHI) | FA News | Google |
| 2011/08/04 | Shake-up looms for hospital managers | Fin24 | Google |
| 2011/08/05 | Sickening prospect | Financial Mail | PressReader |
| 2011/08/05 | Merging for survival | Financial Mail | PressReader |
| 2011/08/05 | Admitted to parliament | Financial Mail | PressReader |
| 2011/08/06 | Qualified CEOs to replace 'clerks' | TimesLIVE | TimesLive |
| 2011/08/08 | Health team planned for each electoral ward | Cape Times | SAM |
| 2011/08/08 | New NHI 'will secure healthier, wealthier future for the masses' | Cape Times | SAM |
| 2011/08/09 | Details of NHI should be known this week | Pretoria News | SAM |
| 2011/08/09 | Details of NHI to be released | City Press | Google |
| 2011/08/10 | Will health tax cost us an arm and a a leg? | Daily Maverick | Google |
| 2011/08/10 | Health tax only way to fund NHI scheme | Timeslive | Google |
| 2011/08/10 | Path to an NHI scheme for SA | Timeslive | Google |
| 2011/08/10 | Here comes the NHI | Bizcommunity | Google |
| 2011/08/11 | Govt treats caregivers poorly | The Herald | PressReader |
| 2011/08/11 | Time for a new attitude | Daily News | SAM |
| 2011/08/11 | Call for probe into courier pharmacies | Business Day | PressReader |
| 2011/08/11 | Operation historic healthcare one step closer, but figures still scanty | Daily Maverick | Google |
| 2011/08/11 | National Health Insurance will require R125 billion in 2012 | TimesLive | Google |
| 2011/08/11 | DA: NHI won't bring quality healthcare | IOL | Google |
| 2011/08/11 | Regulate private healthcare: ANCYL | IOL | Google |
| 2011/08/11 | Audits ahead of NHI pilot | IOL | Google |
| 2011/08/11 | NHI to cost R125bn by next year | City Press | Google |
| 2011/08/11 | NHI contributions will be compulsory | Fin24 | Google |
| 2011/08/11 | Govt ready to buffer economy if necessary | Timeslive | Google |
| 2011/08/12 | Green paper pledges comprehensive package of care | Cape Times | SAM |
| 2011/08/12 | Motsoaledi sets his sights on a revolution | Cape Times | SAM |
| 2011/08/12 | New insurance is no panacea for crumbling health sector, says opposition | Cape Times | SAM |
| 2011/08/12 | All pay and all get care under national health | The Star | SAM |
| 2011/08/12 | Medical aid tax benefits may go | Daily News/ Cape Argus | SAM |
| 2011/08/12 | SA gears for National Health Insurance | Brand SA | Google |
| 2011/08/12 | NHI to be rolled out more slowly | The Star | SAM |
| 2011/08/12 | Tax hike is last resort to fund NHI | Business Day | PressReader |
| 2011/08/12 | NHI clarity needed before we can share Motsoaledi's joy | The Times | Google |
| 2011/08/12 | Hospital bullying and filth under microscope | The Times | Google |
| 2011/08/12 | Green paper reveals costs for next 25 years | The Star | SAM |
| 2011/08/12 | Some welcome NHI, others raise red flag over costs, tax burden | The Star | SAM |
| 2011/08/12 | Good health at fever pitch | The Star | SAM |
| 2011/08/12 | Cosatu 'opposed to multi-payer system' to NHI | Business Day | PressReader |
| 2011/08/12 | Unless state hospitals improve, national insurance will change little | Business Day | PressReader |
| 2011/08/12 | You're the cash cow! | The Star | SAM |
| 2011/08/12 | Health scheme shock | The Herald/ TimesLive | PressReader |
| 2011/08/12 | National Health Insurance: A dummy's guide | Mail & Guardian Online | Google |
| 2011/08/12 | South Africa unveils universal health care scheme | BBC News | Google |
| 2011/08/12 | Factbox - S. Africa's National Health Insurance plan | Reuters | Google |
| 2011/08/12 | Discovery: NHI will boost economy | Fin24 | Google |
| 2011/08/12 | NHI may reduce medical costs | IOL | Google |
| 2011/08/12 | NHI's R125bn price tag for one year | IOL | Google |
| 2011/08/12 | Numsa supports NHI | IOL | Google |
| 2011/08/12 | Cosmetic surgery not covered by NHI | City Press/ TimesLIVE | Google |
| 2011/08/12 | NHI: Private doctors will be crucial | Fin24 | Google |
| 2011/08/12 | NHI a carbon copy of UK's flawed National Health System: iLive | Timeslive | Google |
| 2011/08/12 | NHI costs will rise to R225 billion in 15 years | Bizcommunity | Google |
| 2011/08/13 | Foreboding over NHI well-founded | Weekend Post | PressReader |
| 2011/08/13 | Scheme a threat to economy | Weekend Post | PressReader |
| 2011/08/13 | Make it work | Weekend Witness | SAM |
| 2011/08/13 | Experts examine National Health Insurance draft | Saturday Star | SAM |
| 2011/08/13 | Medical schemes likely to survive until national health system is alive and well | Saturday Star | SAM |
| 2011/08/13 | National Health Insurance: quick facts | City Press | Google |
| 2011/08/14 | NHI paper a symptom of the malaise | Sunday Tribune | SAM |
| 2011/08/14 | Challenges on the road to NHI | Sunday Tribune/ Sunday Independent/ Sunday Argus | SAM |
| 2011/08/14 | More money won't cure ailing health system | Sunday Times | Google |
| 2011/08/14 | Straining under welfare's weight | Sunday Times | PressReader |
| 2011/08/14 | Health companies eye NHI | Sunday Times | PressReader |
| 2011/08/14 | Top hospital bosses may lose jobs | Sunday Times | PressReader |
| 2011/08/14 | Health where it hurts | City Press | SAM |
| 2011/08/14 | NHI funding proposal faces legal challenges | Sunday Times | PressReader |
| 2011/08/14 | So many questions | Sunday Times | PressReader |
| 2011/08/14 | Much to be done before NHI | Sunday Argus | SAM |
| 2011/08/14 | How the NHI scheme will work | Sunday Times | Google |
| 2011/08/14 | NHI to cut in to private care profit | Sunday Independent | SAM |
| 2011/08/14 | NHI is no panacea but it's a remedy | City Press | SAM |
| 2011/08/14 | Medical aids will retain members | City Press | SAM |
| 2011/08/14 | I want to be able to choose'/'The NHI must cover us all'/She wants better services | City Press | SAM |
| 2011/08/15 | Shake up needed for NHI to work | The Herald | PressReader |
| 2011/08/15 | Criminal to allow gross healthcare inequity to continue | Cape Times/ Sunday Independent | SAM |
| 2011/08/15 | A bridge across the wealth gap | Daily News | SAM |
| 2011/08/15 | Costs may see medical aid members drop schemes | The Star | SAM |
| 2011/08/15 | Freeway to financial ruin | The Star/Pretoria News | SAM |
| 2011/08/15 | Is NHI a magic bullet? | Business Day | PressReader |
| 2011/08/15 | SA is still in the dark over NHI | Business Day | PressReader |
| 2011/08/15 | New health staff plan 'key to NHI' | Business Day | PressReader |
| 2011/08/15 | NHI green paper thin on details - industry | The Star | SAM |
| 2011/08/15 | NHI could be 'unconstitutional' | Fin24 | Google |
| 2011/08/15 | NHI puts the cart before the horse | Citizen | SAM |
| 2011/08/15 | The healthcare shakeup! | Citizen | SAM |
| 2011/08/15 | HASA welcomes release of NHI Green Paper | Bizcommunity | Google |
| 2011/08/15 | Discovery Health supports NHI | Bizcommunity | Google |
| 2011/08/16 | Medical aids not impacted on ‘for at least 20 years’ | The Herald | PressReader |
| 2011/08/16 | A 'yes' and 'no' to NHI plan | The Herald | PressReader |
| 2011/08/16 | Medical aid | Cape Times | SAM |
| 2011/08/16 | Which hat will the minister wear for the NHI? | The Star | SAM |
| 2011/08/16 | They should give up medical aid first | The Star | SAM |
| 2011/08/16 | Question mark hanging over future of Gems | The Star | SAM |
| 2011/08/16 | Better structure for better health | The Star | SAM |
| 2011/08/16 | National Health Insurance not a cure at all | The Witness | Google |
| 2011/08/17 | NHI another money-making tool | The Star | SAM |
| 2011/08/17 | New health system will bleed patients dry | Daily News | SAM |
| 2011/08/17 | Arms for education | Citizen | SAM |
| 2011/08/17 | It's simple | Natal Witness | SAM |
| 2011/08/17 | Staff shortage threatens state's health programme | NWU, Potchefstroom Campus, News | Google |
| 2011/08/17 | S.Africa's NHI to bite health insurer's profits | Reuters | Google |
| 2011/08/17 | DA: ANC shutting down debate on the NHI | IOL | Google |
| 2011/08/17 | How the NHI will change schemes | Fin24 | Google |
| 2011/08/18 | The city's health services best in SA | Cape Times | SAM |
| 2011/08/18 | Ethical victory in health care for all | The Star/Cape Times | SAM |
| 2011/08/18 | Health boss tells of security lapses at state hospitals | Business Day | PressReader |
| 2011/08/18 | MP's get glimpse of NHI planning | TimesLIVE | Google |
| 2011/08/18 | NHI is the ANC's latest 419 scam | The Star | SAM |
| 2011/08/18 | Joburg ranks only 20th in audit for health services | The Star | SAM |
| 2011/08/18 | NHI no use without real reform | Mail & Guardian | SAM |
| 2011/08/18 | Mandatory NHI payments on cards | Mail & Guardian | SAM |
| 2011/08/18 | DA unhappy about health committee | Health24 | Google |
| 2011/08/19 | NHI in numbers | Financial Mail | PressReader |
| 2011/08/19 | Cough up for the doc | Financial Mail | PressReader |
| 2011/08/19 | Another costly white elephant | Financial Mail | PressReader |
| 2011/08/19 | For a better health set-up SA needs to keep nurses | Business Day | PressReader |
| 2011/08/19 | DA accuses ANC of muzzling debate on NHI | Bizcommunity | Google |
| 2011/08/19 | My hospital bill | Health24 | Google |
| 2011/08/19 | NHI could sicken economy | Health24 | Google |
| 2011/08/20 | Hospital managers in firing line | Saturday Independent | SAM |
| 2011/08/21 | R1 480 is too much | City Press | SAM |
| 2011/08/21 | I'll pay for their sake | City Press | SAM |
| 2011/08/21 | Will unionised workers fund the NHI | Sunday Times | PressReader |
| 2011/08/21 | NHI prescribes the right medicine | Sunday Times | PressReader |
| 2011/08/21 | How health plan could hit you | City Press | SAM |
| 2011/08/21 | Health technology experts meet to share ideas and devise plans on the safety and quality of medical devices: towards the National Health Insurance | City Press/ Mail & Guardian | SAM |
| 2011/08/21 | Tougher times ahead | Sunday Times | PressReader |
| 2011/08/21 | Well, there goes the cushy ward | TimesLIVE | Google |
| 2011/08/22 | Hospitals fall short | TimesLIVE | TimesLive |
| 2011/08/22 | The NHI unpacked | Bizcommunity | Google |
| 2011/08/22 | Mediclinic welcomes NHI opportunity | The Star | SAM |
| 2011/08/23 | Mammoth task to clear murky picture of public health staff needs | Business Day | PressReader |
| 2011/08/23 | Mediclinic welcomes NHI opportunity | Bizcommunity | Google |
| 2011/08/24 | NHI tax to equal aid scheme costs' | IOL | Google |
| 2011/08/24 | NHI proposals 101 | Health24 | Google |
| 2011/08/24 | Medical aid fees to guide NHI payments | Fin24 | Google |
| 2011/08/25 | Public health care faces big obstacles | The Star | SAM |
| 2011/08/25 | Don't change tyre while car is moving | The Star | SAM |
| 2011/08/25 | NHI will boost service delivery | The Star | SAM |
| 2011/08/25 | NHI payments will be based on medical aid fees | Bizcommunity | Google |
| 2011/08/25 | Making healthcare equitable | Mail & Guardian | SAM |
| 2011/08/25 | Follies the NHI should avoid | Mail & Guardian | SAM |
| 2011/08/25 | Owning the NHI system | Mail & Guardian | SAM |
| 2011/08/26 | Why pay twice? | Financial Mail | PressReader |
| 2011/08/26 | Hands of private medicine in SA | The Star | SAM |
| 2011/08/26 | NHI: History repeats itself | Bhekisisa | Google |
| 2015/11/28 | Pets are family, domestics are workers' | Weekend witness | SAM |
| 2015/12/08 | Gauteng's ambulance fleet boosted by 92 more | The Star | SAM |
| 2015/12/10 | NHI funding proposals keenly watched | Business day | PressReader |
| 2015/12/10 | NHI White Paper to Be Released Tomorrow | eHealth news | Google |
| 2015/12/11 | NHI one step closer | Citizen | SAM |
| 2015/12/11 | National health insurance will be compulsory for all South Africans | Health24/BusinessTech | Google |
| 2015/12/11 | Long-awaited NHI White Paper released | East Cape Radio | Google |
| 2015/12/11 | How South Africa’s NHI will work | News24 | Google |
| 2015/12/11 | One emergency number on the cards – Motsoaledi | News24 | Google |
| 2015/12/11 | White Paper on National Health Insurance released today | Health e-news/ Health24 | Google |
| 2015/12/12 | Healthcare R72bn shortfall | Daily Dispatch | PressReader |
| 2015/12/12 | Medical schemes ‘punish poor’ | The Citizen | Google |
| 2015/12/12 | South Africa needs to spend more on healthcare to achieve universal cover | The Conversation | Google |
| 2015/12/13 | NHI white paper sketchy on costs to SA taxpayers | City Press | SAM |
| 2015/12/13 | Health-E News: NHI White Paper doesn’t explain how it will get buy-in from private doctors | Daily Maverick | Google |
| 2015/12/14 | NHI spells death of medical aids | The Herald | PressReader |
| 2015/12/14 | Health costs dismissed as a thumbsuck | Business day | PressReader |
| 2015/12/14 | NHI White paper lacks details we asked for in 2011 - Economist | Cape Talk | Google |
| 2015/12/14 | White paper: National Health Insurance for South Africa | Health e-news | Google |
| 2015/12/15 | NHI rollout nears its end | Cape Argus | SAM |
| 2015/12/15 | NHI to 'ensure quality healthcare for all' | East Cape Radio | Google |
| 2015/12/15 | NHI White Paper Released | eHealth news | Google |
| 2015/12/17 | All the nuts and bolts of the NHI | Bhekisisa | Google |
| 2015/12/18 | NHI white paper: the good and the bad | GroundUp | Google |
| 2015/12/20 | National Health Insurance | City Press | SAM |
| 2015/12/23 | Big plans for SA’s health | Stanger Weekly | Google |
| 2017/06/17 | NHI could see government paying contractors for Aids treatment | Saturday Argus | SAM |
| 2017/06/21 | ANCYL vows to ensure unemployed KZN doctors are placed in hospitals | Natal Witness | SAM |
| 2017/06/22 | Cheap med aid launched | The New Age | SAM |
| 2017/06/22 | SA a step closer to accessing free healthcare, Cabinet says | News24 | Google |
| 2017/06/23 | NHI white paper approved | The New Age/ BusinessTech | SAM |
| 2017/06/23 | South African cabinet approves White Paper on National Health Insurance as policy document | Africa News Wire | Google |
| 2017/06/23 | Why are they opposing National Health Insurance? | News24 | Google |
| 2017/06/26 | NHI - our new centre of gravity | Cape Times/Pretoria News/ The Star | SAM |
| 2017/06/26 | As the ANC hold its policy conference from Friday, COSATU president S'dumo Dlamini has called on the ruling party to agitate for the rapid implementation of the NHI | Pretoria News/The Star/ Cape Times | SAM |
| 2017/06/27 | Private health-care needs to grow | Hillcrest Fever | Google |
| 2017/06/29 | Makhura to pay NGOs | The New Age | SAM |
| 2017/06/29 | How you’ll be paying for National Health Insurance – more taxes | BusinessTech | Google |
| 2017/06/29 | NHI to cost R69bn over a period of four years – Motsoaledi | The Citizen | Google |
| 2017/06/29 | Motsoaledi says new NHI will bring about radical change | Cape Talk/702 | Google |
| 2017/06/29 | Interns decline placements' - Motsoaledi | Daily Sun | Google |
| 2017/06/29 | Minister releases more details on National Health Insurance | JacarandaFM | Google |
| 2017/06/29 | Health minister uses NHI briefing to reprimand the media | City Press | Google |
| 2017/06/30 | NHI to help women, children and the elderly first | The Times | Google |
| 2017/06/30 | Writing on wall for medical aids | Mail & guardian | SAM |
| 2017/06/30 | Health insurance soon mandatory in SA, funded by tax | Natal Witness | SAM |
| 2017/06/30 | National Health Insurance approved and gazetted | Health24 | Google |
| 2017/06/30 | NHI faces uphill battle ahead | The New Age | SAM |
| 2017/06/30 | R69bn: four-year NHI price tag | Pretoria News | SAM |
| 2017/06/30 | NHI white paper ignores the economic downturn | Business Day | PressReader |
| 2017/06/30 | South Africa issues White Paper on National Health Insurance | Africa News Wire | Google |
| 2017/06/30 | NHI White Paper to be policy document | Africa News Wire | Google |
| 2017/06/30 | Govt promises health cover | Daily Sun | Google |
| 2017/07/03 | The new role of medical aids under the NHI | BusinessTech | Google |
| 2017/07/03 | We're sick of poor working conditions‚ say public sector doctors | DispatchLIVE | Google |
| 2017/07/03 | Let's be honest: state is not capable of implementing NHI | TimesLIVE | Google |
| 2017/07/03 | NHI white paper issues raised | BizCommunity | Google |
| 2017/07/05 | AfroCentric Makes Case for PPPs Under NHI | eHealth news | Google |
| 2017/07/10 | ANC wants your medical aid tax rebate to fund National Health Insurance | BusinessTech | Google |
| 2017/07/10 | Health-e News: Role-players don’t see eye-to-eye on the NHI | Daily Maverick | Google |
| 2017/07/11 | Nod for proposal to fund health plan with medical aid rebates | Business Day | PressReader |
| 2017/07/11 | Health scheme rolled out | Daily Sun | SAM |
| 2017/07/11 | ANC wants medical aid tax rebates to fund the NHI | Natal Witness | SAM |
| 2017/07/12 | NHI Won’t Offer Quick Fix to SA’s Mental Healthcare Crisis | eHealth news | Google |
| 2017/07/13 | Red card for Motsoaledi over his stand on medical-aid tax credits | Business Day | PressReader |
| 2017/07/14 | Fury at plan to can medical aid tax credits | Pretoria News | SAM |
| 2018/06/07 | Medical Aid Industry Must Take Bold Steps Towards UHC | eHealth news | Google |
| 2018/06/08 | NHI is no public health panacea | Business Day | PressReader |
| 2018/06/08 | NHI Bill gets green light for public comment | BusinessLive | Google |
| 2018/06/09 | The real reason why Cosatu wants Motsoaledi's head/Miserable bugger Motsoaledi is good medicine with bad side-effects | Independent on Saturday/The Citizen/ Saturday Argus/Citizen Saturday | Google/ SAM |
| 2018/06/09 | National Health Insurance Bill approved for public comment | eNCA | Google |
| 2018/06/10 | Health minister outlines plan of action | City Press | Google |
| 2018/06/10 | Maybe it's time to get a new health minister | City Press | Google |
| 2018/06/10 | Health standards flatline | Sunday Independent | SAM |
| 2018/06/11 | NHI Bill Approved for Public Comment | eHealth news | Google |
| 2018/06/11 | Cabinet approves an NHI bill that is thin on details | Business Day | PressReader |
| 2018/06/12 | Open clinics 24/7 throughout the week - EFF urges | Pretoria News/Daily News/The Star | SAM |
| 2018/06/12 | Healthcare on life support | The Citizen | SAM |
| 2018/06/13 | NHI pilot project has not worked | Cape Talk/702 | Google |
| 2018/06/13 | Healthcare: no need to reinvent the wheel | Business Day/ e-health news | PressReader |
| 2018/06/13 | Healthcare is in ICU | The Star | SAM |
| 2018/06/14 | #HealthcareCollapse: Public Hospitals have become a death-trap for the poor. Time for drastic action | Democratic Alliance | Google |
| 2018/06/14 | Review NHI, urge 99 medical professionals | Pretoria News/IOL/The Star | Google |
| 2018/06/14 | DA calls for public health inquiry | Business Day | Google |
| 2018/06/15 | Private healthcare heavily subsidised by public' | The Star | SAM |
| 2018/06/17 | Bad roads and health workers’ bonuses are not the health minister’s responsibility | Sunday Times | PressReader |
| 2018/06/18 | Motsoaledi to give the low down on heath insurance on Thursday | DispatchLIVE/  BusinessLIVE | Google |
| 2018/06/18 | Release NHI Bill for benefit of workers and poor – SACP | PoliticsWeb | Google |
| 2018/06/20 | 'Motsoaledi won’t collapse private coverage, but he will harm its quality' | Cape Talk/702 | Google |
| 2018/06/20 | Massive shake-up of health laws ahead of NHI | Health e-news/ Health24 | Google |
| 2018/06/20 | 8 things you need to know about the National Health Insurance bills | Fin24/Witness | Google |
| 2018/06/20 | NEHAWU gives NHI full backing | Afro Voice | SAM |
| 2018/06/20 | Motsoaledi: NHI the land question for the health sector | EWN | Google |
| 2018/06/20 | Pressure mounts to launch the NHI | The Star | SAM |
| 2018/06/21 | NHI a step closer | Algoa FM | Google |
| 2018/06/21 | Tough economy affects wellbeing, membership | Business Day | PressReader |
| 2018/06/21 | What the new National Healthcare Insurance Bill says about who will pay – and where medical aids still come in | BusinessTech | Google |
| 2018/06/21 | ‘Under NHI, the rich will subsidise the poor, the young will subsidise the old’ | EyeWitness News | Google |
| 2018/06/21 | These are the 10 massive medical aid changes you need to know about | BusinessTech | Google |
| 2018/06/21 | These 4 graphs show how many South Africans don’t have medical aid – and the surprising rise of traditional healers | BusinessTech | Google |
| 2018/06/21 | New SA medical aid amendments will change your life | The Citizen | Google |
| 2018/06/21 | NHI implementation ‘will take phase-in approach’ | The Citizen | Google |
| 2018/06/21 | Motsoaledi announces much anticipated health insurance bill | Cape Talk | Google |
| 2018/06/21 | ‘NHI is like building a house with no land on which to put it’ | Cape Talk/702 | Google |
| 2018/06/21 | Support for Motsoaledi’s health NHI proposal | DispatchLIVE | Google |
| 2018/06/21 | Motsoaledi: NHI requires massive legal changes/ NHI 'hurricane' predicted | DispatchLIVE/The Herald | Google |
| 2018/06/21 | Medical aids must foot patients’ bills in full – new bill proposes | DispatchLIVE | Google |
| 2018/06/21 | New era for healthcare in Mzansi! | Daily Sun | Google |
| 2018/06/21 | Impossible to calculate how much NHI will cost - Motsoaledi | Fin24 | Google |
| 2018/06/21 | 'The rich will subsidise the poor' – Motsoaledi outlines universal healthcare plan | News24 | Google |
| 2018/06/21 | New era for healthcare as Motsoaledi wants co-payments abolished | Fin24 | Google |
| 2018/06/21 | New health dawn: Motsoaledi to unveil crucial health bills | News24 | Google |
| 2018/06/21 | NHI gets unpacked today | Afro Voice | SAM |
| 2018/06/21 | Reaction to National Health Insurance (NHI) proposals | Bbrief | Google |
| 2018/06/21 | NHI fund will be mandatory - Motsoaledi | TimesLIVE/DispatchLIVE | Google |
| 2018/06/21 | Motsoaledi announces implementation of #NHIBill | IOL | Google |
| 2018/06/21 | Motsoaledi bullish about fund | The Citizen | SAM |
| 2018/06/22 | NHI is on its way and SA’s health-care system won’t ever be the same again | Biznews | Google |
| 2018/06/22 | 6 important questions about the NHI that remain unanswered | BusinessTech | Google |
| 2018/06/22 | Sanco says proposed new health laws will help the poor | The Citizen | Google |
| 2018/06/22 | Medical aid companies still quiet on Motsoaledi’s amendments | The Citizen | Google |
| 2018/06/22 | 'Rich and poor must be able to use BOTH healthcare systems' - Health Minister | Cape Talk/702 | Google |
| 2018/06/22 | NHI aims to look at costly private health care, poor public health system | Cape Talk | Google |
| 2018/06/22 | Medical training body responds to Health Minister's health care reforms | Cape Talk/702 | Google |
| 2018/06/22 | NHI Bill 'a missed opportunity' - Rural Health Advocacy Project | Cape Talk/702 | Google |
| 2018/06/22 | NHI bill 'step in right direction'- Discovery Health | DispatchLIVE | Google |
| 2018/06/22 | Minister's promise 'access to medical aid for all' | The Star/Cape Times/ Pretoria News/Daily News | SAM |
| 2018/06/22 | Medical aid must pay full cost' | Daily Sun | Google |
| 2018/06/22 | National Health Insurance a step closer to reality | East Cape Radio | Google |
| 2018/06/22 | BHF Supports Progress Made on NHI | eHealth news/ politicsweb | Google |
| 2018/06/22 | New medical schemes Bill intended to “protect” beneficiaries | Health e-news/News24 | Google |
| 2018/06/22 | Major medical schemes support NHI's universal healthcare goal | Fin24 | Google |
| 2018/06/22 | 81% of households that use public healthcare 'satisfied' - Stats SA | News24 | Google |
| 2018/06/22 | Unions welcome new healthcare laws – with a laundry list of concerns | Fin24 | Google |
| 2018/06/22 | 6 ways the NHI bill will affect consumers' pockets | Fin24 | Google |
| 2018/06/22 | Medical aids to be in line with NHI | Afro Voice | SAM |
| 2018/06/22 | 7 massive ways the NHI will affect you – from C-sections to registering with a doctor | Business Insider/ Weekend Witness | Google |
| 2018/06/22 | Problems with the NHI: Why it cannot work | News24 | Google |
| 2018/06/22 | National Health Insurance will 'revolutionise' healthcare, says Sanco | News24 | Google |
| 2018/06/22 | Medical schemes: More money in members' pockets – for now | Health24 | Google |
| 2018/06/22 | Media trivialised a great initiative | Pretoria News | SAM |
| 2018/06/22 | Medical aid fund bombshell | Business Day/ The Herald | PressReader |
| 2018/06/22 | New bill sets out changes to medical aids | Sowetan | PressReader |
| 2018/06/22 | Medical schemes set to soon pay more and take less | Witness | SAM |
| 2018/06/22 | Proposed laws may cost medical aids billions | Mail & Guardian | SAM |
| 2018/06/22 | Everything you need to know about the NHI fund and why it is compulsory | IOL | Google |
| 2018/06/22 | Government Employees Medical Scheme reacts to health bills | TImesLive/Daily Dispatch | Google |
| 2018/06/22 | Medical aid schemes will oppose NHI: Nzimande | eNCA | Google |
| 2018/06/23 | NHI plan would have disastrous consequences – Free Market Foundation | The Citizen | Google |
| 2018/06/23 | Medical aids welcome NHI ‘on initial review’ | The Citizen | Google |
| 2018/06/23 | South Africa cannot afford the proposed NHI – expert | The Citizen | Google |
| 2018/06/23 | Motsoaledi’s pill better be sweet | The Citizen | Google |
| 2018/06/23 | NHI bill proposes doing away with healthcare brokers | Saturday Independent/ Pretoria News Weekend/Saturday Star | SAM |
| 2018/06/23 | Boost for NHI Bill | Pretoria News Weekend/Saturday Star | SAM |
| 2018/06/23 | Cautious nod for proposed NHI Bill | Weekend Post | PressReader |
| 2018/06/23 | Top-class treatment in collaboration | Daily Dispatch | PressReader |
| 2018/06/23 | NHI: Another pipe dream! | Daily Sun | Google |
| 2018/06/24 | Editorial: A step in a healthy direction | City Press | Google |
| 2018/06/24 | NHI brings radical change and critical risks | City Press | Google |
| 2018/06/24 | Health-care bill set to make sweeping changes | Sunday Independent/ Sunday Tribune | SAM |
| 2018/06/24 | Why we need to take the NHI option now | Sunday Independent/ Sunday Tribune/Sunday Argus | SAM |
| 2018/06/24 | AfriForum hits at NHI 'stillborn' proposal | Sunday Independent | SAM |
| 2018/06/24 | What is the National Health Insurance | Sunday Argus/Sunday Independent | SAM |
| 2018/06/24 | Gravity of SA health restructure | Sunday Argus | SAM |
| 2018/06/24 | Cautious reception for Med Aid bill | Sunday Argus | SAM |
| 2018/06/24 | Doctor exodus feared as NHI proposes bold cure | Sunday Times | PressReader |
| 2018/06/25 | Mass emigration of SA doctors a concern following NHI announcement: report | BusinessTech | Google |
| 2018/06/25 | Medical aid schemes respond to NHI | Cape Talk/702 | Google |
| 2018/06/25 | Public comment on NHI Bill welcomed | Afro Voice | SAM |
| 2018/06/25 | Levelling healthcare playing field | Afro Voice | SAM |
| 2018/06/25 | Changes towards healthcare for all | Afro Voice | SAM |
| 2018/06/25 | Let's unite behind a universal health service that caters to all equally | Cape Times | SAM |
| 2018/06/25 | Unclear bill of health | Business Day | PressReader |
| 2018/06/25 | UK’s National Health Service sets bar high as Motsoaledi unveils two bills in SA | Business Day | PressReader |
| 2018/06/25 | Massive nursing shortage in Gauteng leaves sick patients even more vulnerable | The South African | Google |
| 2018/06/26 | Health Market Inquiry suffers chronic delays | The Citizen | Google |
| 2018/06/26 | NHI fees could see mass exodus of doctors – expert | The Citizen | Google |
| 2018/06/26 | I see a bad moon rising | The Citizen | Google |
| 2018/06/26 | Sorry, but the NHI Bill is just not the right medicine | Daily Maverick | Google |
| 2018/06/26 | Massive changes proposed to private medical schemes | Daily Maverick/Health e-news | Google |
| 2018/06/26 | NHI: Anesthesiologists warns of mass exodus of doctors if legislation is implemented recklessly | News24 | Google |
| 2018/06/26 | “NHI won’t happen, bill infringes people’s rights” | Sowetan | PressReader |
| 2018/06/26 | National Health Insurance | Witness | SAM |
| 2018/06/26 | All you need to know about NHI | The Star/Cape Times | SAM |
| 2018/06/26 | Challenges facing healthcare system are structural rather than clinical | Business Day | PressReader |
| 2018/06/26 | Mixed response to national health scheme | Afro Voice | SAM |
| 2018/06/26 | Section27 slams pace of health inquiry | Afro Voice | SAM |
| 2018/06/26 | Brace yourselves for the economic Ebola that is NHI | TimesLive | Google |
| 2018/06/27 | SAMA welcomes aspect of NHI but said many questions still remain | Algoa FM | Google |
| 2018/06/27 | NHI: What next for your medical aid scheme? | News24/Algoa FM | Google |
| 2018/06/27 | 5 things wrong with government’s massive medical aid changes: DA | BusinessTech | GOogle |
| 2018/06/27 | The doctor will NOT see you now | The Citizen | Google |
| 2018/06/27 | SA needs ‘thousands more specialists’ for NHI | The Citizen | Google |
| 2018/06/26 | Private doctors vow to quit SA over National Health Insurance | Cape Argus/IOL | Google |
| 2018/06/27 | More engagements needed on planned NHI‚ anaesthesiology society says | DispatchLIVE | Google |
| 2018/06/27 | NHI just another SOE: DA | East Cape Radio | Google |
| 2018/06/27 | SAMA Raises Questions About NHI Legislation | eHealth news | Google |
| 2018/06/27 | NHI Needs a Massive Reorganisation of the Health System | eHealth news | Google |
| 2018/06/27 | NHI puts R170 billion 'in the hands of one politician' | Health e-news/ Health24 | Google |
| 2018/06/27 | Reckless implementation of NHI ‘may lead to mass exodus of doctors, specialists’ | City Press | Google |
| 2018/06/27 | Medical specialists threaten to emigrate over NHI | The Star | SAM |
| 2018/06/27 | Regulating doctors fees could furhter harm healthcare | The Star/ Pretoria News/Daily News | SAM |
| 2018/06/27 | We want to be on the same side' | Afro Voice | SAM |
| 2018/06/27 | Motsoaledi's NHI Bill needs clarification, says SA Medical Association | Witness | SAM |
| 2018/06/27 | At a glance: What the National Health Insurance means for you & your pocket | Mail & Guardian | Google |
| 2018/06/28 | Please stop chasing our doctors out of SA, TAC pleads | The Citizen | Google |
| 2018/06/29 | NHI is pie in the sky, Minister Motsoaledi | The Citizen | Google |
| 2018/06/29 | South Africa’s universal health care plan falls short of fixing an ailing system/How Motsoaledi’s well-meaning NHI plans fall short | The Conversation/The Citizen/ News24/The Star | Google |
| 2018/06/29 | Bonitas Reacts with Mixed Feelings to New NHI Bills | eHealth news/ Health24 | Google |
| 2018/06/29 | Insufficient capacity for NHI to function | The Herald | Google |
| 2018/06/29 | Will the NHI actually work? | Mail & Guardian | SAM |
| 2018/06/29 | Health Bills' diagnostic value limited | Mail & Guardian | SAM |
| 2018/06/30 | Funding model key to success of NHI | Saturday Star | SAM |
| 2018/07/01 | Minister must make NHI’s public purpose plain | City Press/Witness | Google |
| 2018/07/01 | Clinix Group excited about NHI | City Press | SAM |
| 2018/07/01 | Dr Fix-it or Mr Hyde | Sunday Times | PressReader |
| 2018/07/01 | Doctors still have concerns over NHI proposal | Sunday Independent | SAM |
| 2018/07/02 | R259 billion NHI figure was a guess – we don’t know what it will cost: Motsoaledi | BusinessTech | Google |
| 2018/07/02 | National Health Insurance: It’s not just about the money | Daily Maverick/Health e-news | Google |
| 2018/07/02 | High Aspirations for Universal Healthcare in South Africa | eHealth news | Google |
| 2018/07/02 | Dr Aaron Motsoaledi is clueless – NHI cannot work in SA | News24 | Google |
| 2018/07/02 | Funding figures vital for assessing health fund viability | Business Day | PressReader |
| 2018/07/02 | Finally, the National Health Insurance is here – to fix nothing | Daily Maverick | Google |
| 2018/07/03 | Motsoaledi’s NHI will burden the middle class more than the rich it aims to tax | Sowetan | PressReader |
| 2018/07/04 | SA health fund association anticipates positive report on costs in sector | The Citizen | Google |
| 2018/07/04 | NHI can address SA's biased health-care system | The Post | Google |
| 2018/07/04 | We need National Health Insurance now more than ever | Daily Maverick | Google |
| 2018/07/04 | NHI – what is it? | Greytown Gazette | Google |
| 2018/07/04 | Cosatu comes out to bat for NHI | TimesLive | Google |
| 2018/07/05 | New healthcare plan promises to overhaul South Africa’s massively skewed system | The Conversation | Google |
| 2018/07/05 | Dismal public healthcare is the real crisis | Business Day | PressReader |
| 2018/07/05 | NHI Bill: Welcome but flawed | Spotlight | Google |
| 2019/07/25 | Compact will ensure quality healthcare, says Ramaphosa | Health e-news | Google |
| 2019/07/25 | Ramaphose: presidential health compact will help implement NHI | EWN | Google |
| 2019/07/26 | South Africa’s NHI pilot project shows ‘mixed’ results | BusinessTech | Google |
| 2019/07/26 | Why the NHI is headed for disaster | BusinessTech | Google |
| 2019/07/26 | Zweli Mkhize pins hope on NHI to improve healthcare | Cape Talk/702 | Google |
| 2019/07/26 | We won't allow medical aids to cripple NHI,' Zweli Mkhize | DispatchLIVE/  SowetanLIVE/TimesLIVE | Google |
| 2019/07/26 | Limpopo hospital in dire state: SAHRC finds dirty areas, rotting and expired food | News24 | Google |
| 2019/07/26 | Presidential Health Compact deal to ensure dawn of new system | Pretoria News | Google |
| 2019/07/26 | SAHRC slams Limpopo Health Department | SABC News | Google |
| 2019/07/28 | Get real about this NHI utopia | City Press | SAM |
| 2019/07/29 | Report reveals failings in NHI | Sowetan/The Herald | PressReader |
| 2019/07/29 | NHI's hits and misses | City Press | Google |
| 2019/07/29 | Our viewpoint vague and unconvincing NHI vision | Witness | SAM |
| 2019/07/29 | NHI pilot projects hard to evaluate | Business Day | PressReader |
| 2019/07/29 | Successful NHI implementation requires collaboration | BizCommunity | Google |
| 2019/07/30 | Why SA’s 4 million migrants must be part of universal health plan | The Conversation/The Citizen/The Herald/ Pretoria News | Google |
| 2019/07/30 | Masuku prepares Gauteng’s health system for NHI | Health e-news/The Citizen | Google |
| 2019/07/30 | Can SA rescue its health system? | Witness/City Press | Google/ SAM |
| 2019/07/31 | Cost of new health-care plan a worry | Cape Argus | SAM |
| 2019/07/31 | National Health Insurance: Is this medical aid paving the way to a new era in benefits? | News24/Witness | Google |
| 2019/07/31 | Gauteng Health to prioritize NHI, safety and security | SABCNews | Google |
| 2019/08/01 | Mkhize’s recipe for ruining healthcare | Financial Mail | PressReader |
| 2019/08/02 | The government is rolling the dice on everyone’s health | Business Day | PressReader |
| 2019/08/04 | Gauteng health budget a kickstart towards NHI | Sunday Independent | SAM |
| 2019/08/04 | Healthcare needs radical reimagining | City Press | SAM |
| 2019/08/05 | NHI aims to help everyone | Cape Argus/The Star/ Daily News/ Diamond Fields Advertiser/Mail & Guardian | SAM |
| 2019/08/05 | National Health Insurance scheme will put your health in ANC hands | AfriForum | Google |
| 2019/08/06 | Plans to strengthen healthcare systems | People's Post | Google |
| 2019/08/06 | Mkhize: more sex education for school going youth | EWN | Google |
| 2019/08/06 | The NHI cannot succeed | Voices360 | Google |
| 2019/08/07 | ANC scores one out of seven for promises kept since elections | The Citizen/AfricaCheck | Google |
| 2019/08/07 | NHI 'can't work in shambles' | Daily Dispatch | PressReader |
| 2019/08/07 | Revised NHI Bill raises more questions than gives answers | Medical Brief | Google |
| 2019/08/07 | Health Minister confident in NHI | eNCA | Google |
| 2019/08/08 | NHI Bill tabled in Parliament | IOL/ Algoa FM | Google |
| 2019/08/08 | Five things you need to know about the new National Health Insurance Bill | Bhekisisa/Daily Sun/News24 | Google |
| 2019/08/08 | The end of medical aid as you know it in South Africa | BusinessTech | Google |
| 2019/08/08 | How you will be paying for the NHI, and what happens to your medical aid – everything you need to know | BusinessTech | Google |
| 2019/08/08 | Taxes and more taxes: what to expect from NHI | TimesLIVE | Google |
| 2019/08/08 | Corruption will be a threat to NHI, Mkhize admits | News24/The Citizen | Google |
| 2019/08/08 | Opposition slams NHI Bill as ANC, Cosatu welcome it | The Citizen/News24 | Google |
| 2019/08/08 | Minister Zweli Mkhize releases National Health Insurance Bill | Cape Talk/702 | Google |
| 2019/08/08 | NHI Bill debuts today | Daily Dispatch | PressReader |
| 2019/08/08 | You, National Health Insurance and the changing face of your medical aid | Daily Maverick | Google |
| 2019/08/08 | NHI bill: here’s what you need to know | Power 98.7 | Google |
| 2019/08/08 | ANALYSIS: The National Health Insurance – What it means and the fights we can expect | Bhekisisa/News24 | Google |
| 2019/08/08 | 4 pitfalls on the road to the National Health Insurance | Bhekisisa | Google |
| 2019/08/08 | NHI Bill: Citizens, refugees, inmates, 'designated foreign nationals' and all children to benefit | News24 | Google |
| 2019/08/08 | South Africa puts initial universal healthcare cost at $17 billion | Reuters | Google |
| 2019/08/08 | NHI one step closer to universal coverage | BizCommunity | Google |
| 2019/08/08 | WATCH: Zweli Mkhize announces NHI Bill: How you will be affected (and goodbye medical aid) | The Citizen | Google |
| 2019/08/08 | NHI to be fully functional by 2026 – Health Dept | EWN | Google |
| 2019/08/08 | Private medical aid could disappear by 2026 if NHI Bill becomes law | The South African | Google |
| 2019/08/08 | Personal income tax surcharges and payroll taxes 'will pay for NHI' | TimesLIVE | Google |
| 2019/08/08 | Government to take control of healthcare under NHI: this is how | TimesLIVE | Google |
| 2019/08/08 | Your taxes will increase to fund NHI, leaked copy of bill shows | SowetanLIVE | Google |
| 2019/08/08 | NHI set to create ‘healthier population’ | JacarandaFM | Google |
| 2019/08/09 | Medical schemes industry thrown lifeline | DispatchLIVE | Google |
| 2019/08/09 | Higher taxes to fund NHI | Witness | SAM |
| 2019/08/09 | One health system for all | Witness | SAM |
| 2019/08/09 | IRR gives thumbs-down to 'costly' National Health Insurance | IOL | Google |
| 2019/08/09 | Public urged to add voice to NHI consultation process | EWN | Google |
| 2019/08/09 | Democratic Alliance outline four key problems with the NHI Bill | The South African | Google |
| 2019/08/09 | Institute of Race Relations not a fan of the NHI Bill | The South African | Google |
| 2019/08/10 | Is this the death of medical aids? | The Citizen | Google |
| 2019/08/10 | Parliament welcomes NHI Bill | East Cape Radio | Google |
| 2019/08/10 | Critics scrutinise the NHI Bill | Saturday Star/Saturday Independent | SAM |
| 2019/08/10 | Time not right to realise NHI | Saturday Independent/ Saturday Star/Saturday Argus/Diamond Fields Advertiser | SAM |
| 2019/08/10 | Opposition: SA not in position to roll out NHI | EWN | Google |
| 2019/08/10 | Opposing views on NHI Bill | Saturday Argus | SAM |
| 2019/08/11 | Massive wave of doctors leaving South Africa ahead of the NHI | BusinessTech | Google |
| 2019/08/11 | NHI Bill gets mixed reactions! | Daily Sun | Google |
| 2019/08/11 | NHI will be here in 2026 | City Press | Google |
| 2019/08/11 | NHI plan doesn't inspire confidence | Sunday Tribune/Sunday Argus/Sunday Independent | SAM |
| 2019/08/11 | Time to say 'No!' to NHI Bill | Sunday Argus/Sunday Tribune/Sunday Independent | SAM |
| 2019/08/11 | What the NHI Bill says | City Press | SAM |
| 2019/08/11 | SA's healthcare bill is 'too prescriptive' | City Press | SAM |
| 2019/08/11 | The NHI wrecking ball set to entrench SA’s terminal economic illness | Business Report | Google |
| 2019/08/11 | NHI fears: SA could face a shortage of doctors due to controversial bill | The South African | Google |
| 2019/08/12 | How much more you would have to be taxed to make NHI ‘work’ | BusinessTech | Google |
| 2019/08/12 | New NHI bill will change which hospitals you can use | BusinessTech | Google |
| 2019/08/12 | 7 big questions the NHI bill leaves unanswered | BusinessTech | Google |
| 2019/08/12 | DA to announce its plan to stop NHI Bill from ‘collapsing SA’s health sector’ | The Citizen/George Herald | Google |
| 2019/08/12 | The NHI health plan raises some serious concerns | The Citizen | Google |
| 2019/08/12 | NHI will cut provinces’ role, says Western Cape | Business Day | PressReader |
| 2019/08/12 | Here’s how the NHI will affect you - including new rules about seeing a specialist | Business Insider | Google |
| 2019/08/12 | Flawed National Health Insurance Bill not vital while SA heads to high care | Business Day | PressReader |
| 2019/08/12 | Expect higher taxes to cover NHI scheme | Cape Times/The Star/ Pretoria News | SAM |
| 2019/08/12 | Public invited to comment on new NHI Bill | Diamond Fields Advertiser | SAM |
| 2019/08/12 | No clarity' on controversial bill | Diamond Fields Advertiser | SAM |
| 2019/08/12 | NHI Bill sent to portfolio committee on health | IOL | Google |
| 2019/08/13 | Discovery the punchbag in health sell-off | The Star/Cape Times/ Pretoria News | SAM |
| 2019/08/13 | Discovery share price hit by National Health Insurance | BusinessTech | Google |
| 2019/08/13 | Discovery on the NHI and future role of medical schemes | BusinessTech | Google |
| 2019/08/13 | You could lose as much as R12,000 a year from the NHI taking your medical aid tax credits | BusinessTech | Google |
| 2019/08/13 | DA questions constitutionality of the NHI Bill | News24/The Citizen/Daily Sun | Google |
| 2019/08/13 | 'The NHI has no clear coherent public purpose,' says pundit | Cape Talk/702 | Google |
| 2019/08/13 | DA heading to court to challenge NHI bill | Cape Talk | Google |
| 2019/08/13 | Discovery closes near five-year low as plunge continues | Business Day | PressReader |
| 2019/08/13 | DA leader Mmusi Maimane challenges the legality of NHI Bill | DispatchLIVE | Google |
| 2019/08/13 | NHI far worse than Zuma’s craziest idea | Business Day/ The Herald | PressReader |
| 2019/08/13 | NHI: Here’s what the new healthcare system could cost the taxpayer | The South African | Google |
| 2019/08/13 | NHI South Africa: Plan to fight state take-over of doctors expected today | The South African | Google |
| 2019/08/13 | SA’S NHI BILL NOT PROPERLY THOUGHT THROUGH, SAYS DOCTORS’ FORUM | EWN | Google |
| 2019/08/13 | SAPPF vetoes proposed NHI model | BizCommunity | Google |
| 2019/08/13 | DA to fight NHI bill 'all the way to the Constitutional Court' | TimesLive | Google |
| 2019/08/13 | Concerns that NHI model will worsen state of health care in the country | The Mercury | Google |
| 2019/08/13 | NHI having a negative impact on the economy - Freedom Front Plus | Business Report | Google |
| 2019/08/13 | NHI already having a negative impact on the economy - FF Plus | PoliticsWeb | Google |
| 2019/08/13 | NHI does not seem to pass constitutional muster – Mmusi Maimane | PoliticsWeb | Google |
| 2019/08/13 | SA Private Practitioners Forum raises concerns about NHI | eNCA | Google |
| 2019/08/14 | The NHI is not a punishment: health minister | BusinessTech | Google |
| 2019/08/14 | The role of private hospitals and doctors under the new NHI | BusinessTech | Google |
| 2019/08/14 | National Hell Insurance vs sustainable national health | The Citizen | Google |
| 2019/08/14 | ‘We’ll turn our economy around, whether people like it or not’ – Ramaphosa | News24/The Citizen | Google |
| 2019/08/14 | SA needs more time, better economy for NHI – experts | The Citizen | Google |
| 2019/08/14 | ANC takes on DA over criticism of NHI Bill | News24/The Citizen | Google |
| 2019/08/14 | Mkhize ‘absolutely not concerned’ about DA’s threat to take NHI Bill to Concourt | The Citizen/News24 | Google |
| 2019/08/14 | NHI will improve health access to all South Africans' | Cape Talk/702 | Google |
| 2019/08/14 | Hard to imagine NHI ever being successful | The Herald | PressReader |
| 2019/08/14 | Ambitious National Health Insurance plan stalked by the shadow of State Capture | Daily Maverick | Google |
| 2019/08/14 | Government taking care of our health? You must be joking | Witness/ News24 | Google |
| 2019/08/14 | DA vows to fight NHI Bill 'to end' | The Star/Cape Argus/ Daily News | SAM |
| 2019/08/14 | NHI feels like an ill-considered choice | The Star/Cape Argus/ Daily News | SAM |
| 2019/08/14 | Need for a balanced healthcare | Cape Times | SAM |
| 2019/08/14 | Worries on how NHI will affect care | Daily Dispatch | PressReader |
| 2019/08/14 | DA wants NHI Bill in line with Constitution | Pretoria News | SAM |
| 2019/08/14 | DA puts up fight against health bill | Daily Sun | SAM |
| 2019/08/14 | DA vows to challenge NHI Bill | Diamond Fields Advertiser | SAM |
| 2019/08/14 | Maimane and DA to challenge legality of National Health Insurance | Sowetan | PressReader |
| 2019/08/14 | National Health Insurance Bill will depress confidence and growth further | Daily Maverick | Google |
| 2019/08/14 | Five reasons why the DA is against the National Health Insurance bill | TimesLIVE | Google |
| 2019/08/14 | Mkhize calls for calm following opposition to the NHI Bill | EWN | Google |
| 2019/08/14 | President Ramaphosa stands by heavily criticised NHI Bill | The South African | Google |
| 2019/08/14 | NHI is here to stay: Cyril Ramaphosa | Daily Dispatch/ SowetanLIVE | Google |
| 2019/08/14 | Institutions dispute claims over NHI doctor exodus | eNCA | Google |
| 2019/08/15 | Making sense of the NHI and your future medical costs – money expert Dawn Ridler | BizNews | Google |
| 2019/08/15 | 6 things that every South African should know about the NHI – according to a legal expert | BusinessTech | Google |
| 2019/08/15 | EXPLAINED: When will the NHI be implemented? | News24/The Citizen/ George Herald | Google |
| 2019/08/15 | DA says parliament has agreed to scrutinise the legality of new NHI laws | The Citizen | Google |
| 2019/08/15 | State attorney to brief health committee on NHI Bill’s constitutionality | News24/The Citizen | Google |
| 2019/08/15 | Opinion on health bill divided in parliament | Cape Argus/The Star | SAM |
| 2019/08/15 | NHI closer to fruition, says health minister | Health e-news | Google |
| 2019/08/15 | Ferial Haffajee: How NHI will send your taxes rocketing | Fin24 | Google |
| 2019/08/15 | New NHI Bill lauded, decried as disastrous | Daily News/The Star/ Cape Argus/Diamond Fields Advertiser | SAM |
| 2019/08/15 | Universities are key to NHI plan | The Star/Pretoria News | SAM |
| 2019/08/15 | NHI will be implemented in a responsible, affordable manner | Cape Times/Pretoria News | SAM |
| 2019/08/15 | A simple breakdown of how the NHI will affect you in the near future | Cape Times | SAM |
| 2019/08/15 | NHI rethink is needed | Business Day | PressReader |
| 2019/08/15 | Cloud bursts on Discovery | Financial Mail | PressReader |
| 2019/08/15 | The fatal flaws of NHI | Financial Mail | PressReader |
| 2019/08/15 | Parliament to study legality of NHI Bill | Business Day | PressReader |
| 2019/08/15 | Board of Healthcare Funders gives NHI thumbs up | EWN | Google |
| 2019/08/15 | NHI prompts decline in private healthcare share price | SABC News | Google |
| 2019/08/16 | Discovery supportive of NHI but limiting role of medical schemes counter-productive | BizNews | Google |
| 2019/08/16 | Discovery responds to the NHI: medical aids aren’t going anywhere | BusinessTech | Google |
| 2019/08/16 | NHI will require tech and data to be a success | The Star/Pretoria News/Cape Times | SAM |
| 2019/08/16 | NHI Bill is not what the ICU doctor ordered | Daily Maverick | Google |
| 2019/08/16 | National Health Insurance will kill smaller medical schemes’ | Financial Mail | PressReader |
| 2019/08/16 | Treating NHI as a battlefield helps no-one | Business Day | PressReader |
| 2019/08/16 | State Attorney to be consulted on Bill | Cape Argus | SAM |
| 2019/08/16 | NHI: the good, the bad and the ugly | Mail & Guardian | SAM |
| 2019/08/16 | Restrictions counterproductive, says Discovery | Business Day | PressReader |
| 2019/08/16 | Limiting role of medical schemes would be counterproductive to NHI - Discovery | Business Report | Google |
| 2019/08/16 | NHI funding identified: Mkhize | SABC News | Google |
| 2019/08/17 | Everyone, including the poor, ‘will cough up for NHI’ | The Citizen | Google |
| 2019/08/17 | Dipping into state savings for NHI a risky gamble, pundits say | The Citizen | Google |
| 2019/08/18 | NHI Bill must prioritise people, not private interest | Health e-news/Daily Maverick | Google |
| 2019/08/18 | Relax, NHI will not happen' | Sunday Tribune/Sunday Argus/Sunday Independent | SAM |
| 2019/08/18 | Government says there is no threat to private practitioners | Sunday Independent/ Sunday Argus | SAM |
| 2019/08/18 | NHI can be sustainable, if we want it to be | Sunday Independent/ Sunday Argus | SAM |
| 2019/08/18 | How NHI scheme will operate | Sunday Tribune | SAM |
| 2019/08/18 | Selected NHI model will only do harm | Sunday Independent/ Sunday Tribune | SAM |
| 2019/08/18 | NHI is a good idea, but must be forged by consultation | Sunday Times | PressReader |
| 2019/08/18 | MEC says NHI will be excellent | Sunday Independent | SAM |
| 2019/08/19 | Why medical aids in South Africa could be around for longer than you think: legal expert | BusinessTech | Google |
| 2019/08/19 | Arguments for and against NHI | News24/The Citizen | Google |
| 2019/08/19 | Why South Africa’s plans for universal healthcare are pie in the sky | EWN/ The Conversation/Daily Maverick/BizNews/ Witness | Google |
| 2019/08/19 | Nicholas Crisp: A man on a mission to make NHI work | Daily Maverick | Google |
| 2019/08/19 | Discovery move to shore up share price | Cape Times/The Star/ Pretoria News | SAM |
| 2019/08/19 | Universal health care can be successful, but conditions in SA make that unlikely | Business Day | PressReader |
| 2019/08/20 | Will it be compulsory for you to join the NHI fund? | Cape Argus/Daily News | SAM |
| 2019/08/20 | ‘Private healthcare not all rosy’ – Gauteng Health MEC Bandile Masuku | News24/The Citizen | Google |
| 2019/08/20 | Healthcare for all is the wish and NHI the means yet to be perfected | Business Day | Google |
| 2019/08/20 | Is the National Health Insurance Bill open to a constitutional challenge? | Business Maverick | Google |
| 2019/08/20 | Neglect at hospitals a threat to NHI | Sowetan | PressReader |
| 2019/08/20 | The NHI and its aspirations for universal healthcare: Points of contention | Daily Maverick | Google |
| 2019/08/20 | Good medicine for some, bitter pill for others: the NHI era has dawned | Personal Finance | Google |
| 2019/08/20 | Reaction to the NHI bill is ill-informed and a constructive discussion is needed | IOL/ Daily News/The Star/Pretoria News/ Cape Times/Cape Argus/Citizen | Google |
| 2019/08/21 | Ideology blinding some to merits of NHI | Business Day | PressReader |
| 2019/08/21 | Discovery’s red flags | Business Maverick | Google |
| 2019/08/21 | Forget NHI: Strengthen what we have, and build what we need — jobs | Daily Maverick | Google |
| 2019/08/21 | The NHI Bill is ‘not doom and gloom’ | Mail & Guardian | Google |
| 2019/08/21 | Migrants and their children to get access to NHI, says Gauten Health MEC | News24/Daily Sun | Google |
| 2019/08/22 | Special advisor to the president answers 6 burning questions about the new NHI in South Africa | BusinessTech | Google |
| 2019/08/22 | To have or not to have an NHI | Daily Maverick | Google |
| 2019/08/22 | Government 'irrevocably committed' to NHI, Cyril Ramaphosa tells Parliament | IOL | Google |
| 2019/08/22 | The National Health Insurance Bill is a blueprint for disaster | Daily Maverick | Google |
| 2019/08/22 | Ramaphosa: We will run a 'clean shop' in our NHI Fund | Fin24 | Google |
| 2019/08/22 | Opponents of NHI are against transformation - Ramaphosa | News24 | Google |
| 2019/08/22 | NHI a chance to fundamentally transform SA’s healthcare system - Ramaphosa | EWN | Google |
| 2019/08/22 | Provinces ask for caution on national health | Business Day | PressReader |
| 2019/08/22 | An alternative to NHI: Unpacking the Sizani Universal Healthcare plan | The South African | Google |
| **Key:** African National Congress (ANC), African National Congress Youth League (ANCYL), Congress of South African Trade Unions (COSATU), Democratic Alliance (DA), Economic Freedom Fighters (EFF), Freedom Front Plus (FFP), Hospital Association of South Africa (HASA), Intensive care unit (ICU), Kwa-Zulu Natal (KZN), Member of the Executive Council (MEC), Minister of Parliament (MP), National Education, Health and Allied Workers’ Union (NEHAWU), Non-governmental organisation (NGO), Public-private partnerships (PPP), South African Human Rights Commission (SAHRC), South African Media (SAM), South African Medical Association (SAMA), South African National Civic Organisation (SANCO), South African Private Practitioners Forum (SAPPF), State-owned enterprise (SOE), Treatment Action Campaign (TAC) | | | |
